# New Pharmacokinetic and Microbiological Prediction Equations to Be Used as Models for the Search of Antibacterial Drugs

**DOI:** 10.3390/ph15020122

**Published:** 2022-01-20

**Authors:** Jose I. Bueso-Bordils, Gerardo M. Antón-Fos, Antonio Falcó, Maria J. Duart, Rafael Martín-Algarra, Pedro A. Alemán-López

**Affiliations:** 1 Departamento de Farmacia, Universidad Cardenal Herrera-CEU, CEU Universities C/Ramón y Cajal s/n, Alfara del Patriarca, 46115 Valencia, Spain; ganton@uchceu.es (G.M.A.-F.); mduart@uchceu.es (M.J.D.); rmartin@uchceu.es (R.M.-A.); paleman@uchceu.es (P.A.A.-L.); 2 ESI International Chair@CEU-UCH, Departamento de Matemáticas, Física y Ciencias Tecnológicas, Universidad Cardenal Herrera-CEU, CEU Universities San Bartolomé 55, Alfara del Patriarca, 46115 Valencia, Spain; afalco@uchceu.es; 3 Departamento de Matemáticas, Física y Ciencias Tecnológicas, Universidad CEU Cardenal Herrera, CEU Universities, San Bartolomé 55, 46115 Alfara del Patriarca, Spain

**Keywords:** pharmacokinetics, antibacterial, multilinear regression (MLR), computational chemistry, molecular topology, molecular connectivity, topological indices, quinolones, QSAR

## Abstract

Currently, the development of resistance of *Enterobacteriaceae* bacteria is one of the most important health problems worldwide. Consequently, there is a growing urge for finding new compounds with antibacterial activity. Furthermore, it is very important to find antibacterial compounds with a good pharmacokinetic profile too, which will lead to more efficient and safer drugs. In this work, we have mathematically described a series of antibacterial quinolones by means of molecular topology. We have used molecular descriptors and related them to various pharmacological properties by using multilinear regression (MLR) analysis. The regression functions selected by presenting the best combination of a number of quality and validation metrics allowed for the reliable prediction of clearance (CL), and minimum inhibitory concentration 50 against *Enterobacter aerogenes* (MIC50Ea) and *Proteus mirabilis* (MIC50Pm). The obtained results clearly reveal that the combination of molecular topology methods and MLR provides an excellent tool for the prediction of pharmacokinetic properties and microbiological activities in both new and existing compounds with different pharmacological activities.

## 1. Introduction

The increasing emergence of resistance to known antibiotics is one of the most important problems that have appeared in recent years in the treatment of infectious diseases nowadays [1]. The emergence of resistance is associated not only with complications from infections in terms of morbidity and mortality, but also represents an increase in healthcare costs [2]. In this context, rapid and inexpensive methods are necessary for the immediate expansion of the therapeutic arsenal that we currently have [3].

Traditionally, in order to develop a drug, it was necessary to synthesize and evaluate the activity of hundreds to thousands of compounds with the intention of proving their biological activity, selectivity, and bioavailability, as well as their low toxicity. On average, this process of discovering new drugs involves, in addition to a high economic cost, several years of duration before, hopefully, finding a drug with the right characteristics to boost its commercialization [4]. 

Virtual screening along with combinatorial chemistry appear as a solution to this problem by allowing researchers to identify molecules that are likely to be active from a virtual chemical library [5]. Given that these virtual libraries usually have many components, it is common to apply filters to discard molecules with a low probability of becoming a drug. In this sense, Lipinski was the first to apply these filters when describing the “Rule of Five” [6]. This standard predicts that a compound is “non-pharmacological” if it has too many hydrogen bond donors or acceptors (5 and 10 respectively), its molecular mass is greater than 500 and its calculated partition coefficient (ClogP) is greater than 5. With these rules, Lipinski created the first pre-filter prior to drug screening [7].

This rule has been revised based on data obtained from studies in rats [8]. Since then, other rules have appeared and been modified, such as the Rule of Three [9], used in “fragment-based” discovery, which classifies fragments as “drug-like” those with an average molecular weight ≤ 300 Da, a calculated partition coefficient (ClogP) ≤ 3, ≤3 hydrogen bond donors, ≤3 hydrogen bond acceptors, and <3 rotatable bonds. Another similar example is the “Rule of 3/75”, which was described after analyzing 245 preclinical Pfizer compounds. This rule states that a compound will be 2.5 times safer in in vivo assays when its ClogP is <3 and the topological polar surface area (TPSA) > 75 [10]. 

Within drug screening, it is also necessary to highlight the rise of QSAR (Quantitative Structure-Activity Relationship) methods, which have been the foundation of the design and development of new molecules with pharmacological activities [11]. QSAR modelling is a technique that allows the interdisciplinary exploration of knowledge in compounds covering chemical, physical, biological, and toxicological aspects. It also provides formalisms for the mathematical development of models based on chemical characteristics and the activity of structurally similar compounds. This context is defined by mathematical algorithms and provides a reasonable foundation for creating a prediction model [12].

The use of QSAR methods for obtaining and developing new drugs is a key tool since it provides a noteworthy saving of time and resources, considering that many pharmacological activities of compounds can be predicted without having been synthesized by virtue of its rational approach [13,14].

There are numerous examples of drug designed molecules with very different pharmacological activities which have followed the application of QSAR techniques in combination with other in-silico methods, such as zanamivir, tirofiban, imatinib, raltegravir, donepezil, boceprevir, norfloxacin, captopril and dorzolamide, all of them approved by the US Food and Drug Administration [15].

Our research group uses molecular connectivity or molecular topology (MT), developed by Kier and Hall in the mid-1970s [16], a method derived from the QSAR techniques, which is based on the calculation of molecular descriptors or topological indices (TIs) from the structure of a molecule. These methods play an important role, since they provide useful information for the rational design of new molecules with minimal cost and are an alternative and effective tool in view of the reduction of investments by the pharmaceutical industry in the area of antibiotics [17,18].

By using MT, we can classify a compound as active or not active against a certain pharmacological activity using pattern recognition techniques such as multilinear regression (MLR), discriminant linear analysis, neuronal networks, random forests, support vector machines or principal component analysis [19].

All Gram-negative bacilli of the *Enterobacteriaceae* family, are resistant to common antimicrobials, such as penicillin, oxacillin, methicillin, macrolides, glycopeptides, linezolid, and clindamycin, among others [20]. Moreover, the World Health Organization (WHO) on its “WHO PRIORITY PATHOGENS LIST FOR R&D OF NEW ANTIBIOTICS” considers this family a critical or first priority pathogen [3]. Both bacteria studied in this work, *Proteus mirabilis* and *Enterobacter aerogenes*, are Gram-negative bacilli that belong to the *Enterobacteriaceae* family, and are an important cause of community and nosocomial infections, representing a major clinical and public health challenge due to the limited therapeutic arsenal and well-established resistances [21,22].

In this study, by combining MT and MLR after extending a thorough bibliographic research on physicochemical and biological properties on antibacterial quinolones from a previous study [23], we developed three linear regression functions capable of predicting clearance and minimum inhibitory concentration 50 against *Enterobacter aerogenes* and *Proteus mirabilis*. We selected quinolones to build these equations, which can be applied to other quinolones not used in the study, as well as to molecules that share no structural relationship, seeing as the selection of hits or leads considers mathematical-topological similarity patterns, despite the possible core-structure diversity [24]. Thus, the obtained functions can be used as filters in the search process of new antibacterials in large databases of molecules. Examples of some commonly used chemical libraries where new molecules could easily be found on which to apply these techniques are ZINC, Benchmark DUD, PubChem, ChemBank, ChEMBL, DrugBank and Inter-bioscreen [15].

The first quinolone to be used clinically was discovered in 1962 by G. Lesher et al., who, during the purification of the antimalarial chloroquine from mother liquor, found 7-chloroquinoline, a by-product with antimicrobial activity. Starting from this lead, nalidixic acid was obtained. Despite the good tolerance and ease of synthesis of this compound, its use has been restricted to the treatment of some urinary infections due to its tendency to select resistance and to concentrate mainly in urine. From this discovery, derivatives with similar structures were developed, with still moderate activity against Gram-negative bacteria, but which were very valuable in uncomplicated urinary infections [25].

Pipemidic acid appeared in 1975, the first quinolone with a piperazine ring, with favorable activity, spectrum, and pharmacokinetics, which allowed the administration of lower doses. The appearance of bacterial resistance during treatment and side effects were also lower [26]. A year later, flumequin was discovered, which, by incorporating a fluorine atom in position 6 of the core double ring, was able to amplify its spectrum within *Enterobacteriaceae*, including some resistant strains and slightly improve the activity against Gram-positive bacteria [27].

These facts motivated the beginning of an active chemical synthesis campaign to refine the structure-activity relationships. The aim was to improve activity while optimizing the pharmacokinetic properties and reducing the toxicity and interactions with other drugs. This led to the consolidation of the second-generation quinolones, which differ from the classical quinolones in two characteristics common to all of them: the presence of a fluorine atom in position 6 and a substituted piperazine or pyrrolidine ring at position 7 of their core structure [28].

The first second-generation quinolone to be patented, in 1978, was norfloxacin, whose improved potency against Gram-negative bacteria was already in the natural antibiotics’ range, in addition to having a longer half-life (3–4 h) and shorter protein binding (50%) than its predecessors [29]. Shortly after, ciprofloxacin was introduced, being for many years the most potent fluoroquinolone against Gram-negative bacteria and the most widely used quinolone [30]. This generation of quinolones has a much higher activity against aerobic Gram negatives and some important Gram-positive pathogens, as well as more favourable toxicological profiles and good absorption through the gastrointestinal tract [28].

In the 1990s, several quinolones, mostly di- or tri-fluorinated, were approved. They are the third-generation quinolones, which have a greater structural complexity than their predecessors. One of the first modifications was the introduction of an amino group at position 5, which caused an increase in the general activity against Gram-positive bacteria [28]. All of them have advantages in their bioavailability compared to second-generation quinolones, since the substituted rings at position 7 give them greater lipophilicity, resulting in longer elimination half-lives and greater tissue penetration [31].

In recent years, a new class of quinolones has been synthesized, since the optimization of the substituents in different positions of the core structure has allowed the suppression of the fluorine atom in position 6, thus avoiding possible toxic effects, without necessarily reducing its activity [32]. Probably, the most prominent examples of these novel des-fluoro(6) quinolones are garenoxacin (initially known as BMS-284756) and DX-619, which due to their high affinity for type II topoisomerases, have shown high intrinsic activity against most Gram-negative, Gram-positive and anaerobic microorganisms with a low resistance selection potential [33,34].

The good pharmacokinetic profile of quinolones, as well as their high antibacterial activity and broad spectrum of action, makes their therapeutic indications very varied. In fact, quinolones are used in the treatment of many infections, both in the hospital setting and in outpatient clinical practice. More than ten thousand quinolones have been registered. Therefore, it has probably been the most widely used thoroughly studied class of chemotherapeutic agents [35,36]. For all these reasons, we thought that quinolones were the most suitable antibacterial family to be the focus of our study.

QSAR models have been widely attempted, using homologous and congeneric series of active compounds, obtaining excellent results for the correlation of fluorescent [37], antitumor [38], anti-HIV [39], antifungal [40] and antibacterial [41,42] properties, amongst others.

## 2. Results

We performed Y-randomization, inter-correlation, and leave-one-out (LOO) cross-validation tests on all equations. On the one hand, a representation of correlation coefficient (*r*^2^) versus cross-validated correlation coefficient (*r*^2^*_cv_*) could show the presence of chance correlations. On the other hand, compounds that do not fit the selected function could be easily spotted by a representation of the Y-randomization analysis. 

Hence, we rejected those equations with highly dependent indices (correlation coefficient greater than 0.8 or smaller than −0.8 between indices), suspected randomness (*r*^2^*_vc_* smaller than 0.5 for the selected equation) or outliers in the compound set (dissimilar residual values between the selected and the cross-validated equation for one of the compounds, which is indicative of instability). 

We also considered adjusted linear coefficient of determination (*r*^2^*_a_*), which is represented by:

*r*^2^*_a_* = *r*^2^ − (*p*(*1* − *r*^2^)/(*N* − *p*′))

where *N* is the number of observations; and *p* is the number of dependent variables selected in the RF (*p′* = *p* when the *y*-axis intersect is zero and *p′* = *p* + 1 when it is different from zero). The correlation will be better the closer this value gets to unity.

Therefore, we finally selected three descriptor-independent regression functions (RFs) with high predictive capacity and significant statistical parameters:RF_CL_ = 8981.08 + 1596.68^6^*χ_ch_* − 109.481*S_>N-_* − 2637.68*NI*_2_(1)*n* = 12r = 0.95939F = 30.85*p* = 0.0001SEE = 65.42953r^2^ = 0.92043r^2^_a_ = 0.89059r^2^_cv_ = 0.79791RF_MIC50Ea_ = 0.410929 − 0.123037*S_-CH_*_2*-*_ + 0.0685818*S_aCa_* + 0.0569394*S_-NH-_*(2)*n* = 13r = 0.95930F = 34.62*p* = 0.0000SEE = 0.033071r^2^ = 0.92025r^2^_a_ = 0.89367r^2^_cv_ = 0.83270RF_MIC50Pm_ = 0.376001 + 27.6676^9^*χ^V^_ch_* + 0.0650158*S_-CH_*_3_ − 0.0889109*S_>N-_*(3)*n* = 16r = 0.91837F = 21.54*p* = 0.0000SEE = 0.076808r^2^ = 0.84340r^2^_a_ = 0.80425r^2^_cv_ = 0.70282

In Table 1, Table 2 and Table 3 we can find all the important information regarding the RFs for each of the compounds: the value of each independent variable or TI; the dependent variable or experimental value for each property; the predicted value for each property; and the residual values (difference between experimental and predicted value). 

We can note that one of the calculated values is negative (Clinafloxacin, Table 2) and, of course, this is an impossible value for a MIC value. This fact can happen in compounds with a dependent variable’s value very close to zero, since the linear regression function may calculate a predicted value for that compound just below the *x*-axis, i.e., just below zero.

## 3. Discussion

Only those functions with a significance greater than 99.99% (*p* ≤ 0.0001) were selected. The incorporation of more than three independent variables was studied in order to improve the statistical parameters but this was not achieved with any of the properties studied. Finally, the best statistical data were obtained with the presence of three independent variables in each function, which indicates that all the indices provide important structural information to quantify each of the properties studied.

In all cases, the residual value for each compound was less than twice the standard error of estimation (±2SEE), indicating that the selected equations have high predictive ability.

The cross-validated correlation coefficient (*r*^2^*_cv_*) was determined to study Y-randomization. A function is considered to be non-randomly obtained when its *r*^2^*_cv_* > 0.5 [15]. In the three selected equations, the value of this coefficient was higher than 0.7028, which explains more than 70.28% of the variance of the group. The probability of finding a random sequence that fits the prediction model is very low since in the three selected equations they had significantly higher *r*^2^*_cv_* values than the non-selected equations (Supplementary Section S1). All these data guarantee the robustness of the selected equations.

For the clearance (CL) property, a connectivity index [43], which considers sixth order chain type graphs (^6^*χ_ch_*), increases its value. This means that the presence of 6-member cycles increases this property. One the other hand, an electro-topological index (*S_>N-_*) [44] and an information-theory index (*NI*_2_) [45] have a negative effect on the RF’s value. The first one increases with a high number of tertiary amine groups, so the presence of this group decreases the CL value. The latter can be applied to a molecule to quantify its atomic diversity [46].

For the minimum inhibitory concentration 50 against *Enterobacter aerogenes* (MIC50Ea), two electro-topological indices increase its RF’s value. The first one (*S_aCa_*) increases with a high number of aromatic carbons, while the second one (*S_-NH-_*) increases with a high number of secondary amine groups. Thus, it should be noted that the presence of aromatic carbons and/or secondary amine groups have a negative effect on the activity against *E. aerogenes*, since the property in this case is MIC. On the contrary, another electrotopological index (*S_-CH_*_2-_), which increases with an increasing number of methylene groups, helps decreasing the RF’s value and, hence, improves the activity against *E. aerogenes*.

For the minimum inhibitory concentration 50 against *Proteus mirabilis* (MIC50Pm), a connectivity index and an electro-topological index increase its RF’s value. The valence-connectivity index [47] considers ninth order chain type graphs (^9^*χ^V^_ch_*), present in garenoxacin and moxifloxacin, which present weak activity against *P. mirabilis* compared to most of the compounds in that data set. This is consistent with the structure-activity relationships established by Domagala et al. [48], which state that a 9-member bicycle at the quinolone’s R7 position enhances activity against Gram-positive bacteria but is detrimental for Gram-negative bacteria such as *P. mirabilis*. Since this bicycle is present in only two compounds, the possibility of developing the RF ignoring this index was studied, but in all the attempts, the statistical consistence significantly decreased. The connectivity index’s value (*S_-CH_*_3_) increases with an increasing number of methyl groups, so we could state that an excessive amount of methyl groups in the quinolones’ structure decreases the activity against *P. mirabilis*. In fact, most of the most active compounds from this data set lack this functional group, i.e., have a zero value for this index (see Table 3 for the MIC, ^9^*χ^V^_ch_* and *S_-CH_*_3_ values and Supplementary Section S2 for the quinolones’ chemical structures). Nevertheless, an electro-topological index decreases the RF’s value, i.e., enhances activity against *P. mirabilis*. Such is the case of *S_>N-_*, which increases with a high number of tertiary amine groups. Therefore, we could state that a good proportion of tertiary amine groups in the quinolones’ structure enhances the activity against *P. mirabilis*. 

## 4. Materials and Methods

### 4.1. Compound Selection

Data were collected for a total of 85 physicochemical, pharmacokinetic, pharmacodynamic and microbiological properties of antibacterial quinolones (see Supplementary Section S2).

Experimental procedures for the obtention of experimental values of each property were studied. To select a property, it had to have been obtained with comparable experimental procedures for at least 10 compounds. Sufficient data for a total of 27 microbiological properties (activities against different microorganisms measured as different MICs) were obtained from in vitro assays performed according to CLSI protocols [49] and 9 pharmacokinetic properties obtained in assays performed in healthy subjects.

Due to the diversity of bibliographical data found regarding a given property for each of the selected molecules, we considered the average of those data whose values were of the same order of magnitude for the study and development of the prediction equations.

### 4.2. Topological Descriptors

A total of 41 different quinolones were topologically characterized using DESMOL13 [50] and MOLCONN-Z [51] programs. We removed indices with value 0 for every compound and with identical values for all compounds, leaving a total of 140 topological descriptors specific to each molecule (see Supplementary Section S3). These descriptors were computed from the adjacency topological matrix obtained from the hydrogen-depleted chemical pseudographs, drawn with the ChemBioDraw Ultra 12.0 drawing program of the ChemBioOffice 2010 program package. The molecular descriptors used are described in Supplementary Section S4 along with their definitions and references. 

### 4.3. Multilinear Regression

We used the statistical package BMDP module 9R [52] to perform the MLR analysis. This program is based on the Furnival-Wilson algorithm [53], which uses Mallow’s Cp parameter [54] to assess how well a set of observations fit a given RF. Its purpose is to find the best combination of indices from all the possible ones to generate this RF. This statistical parameter can be depicted as:

*Cp* = *PRESS*/*s*^2^ − (*N* − 2*p*)

where *PRESS* is the squared sum of the residual values of the dependent variables; *s* is the residual mean obtained in the RF; *p* is the number of dependent variables selected in the RF; and *N* is the number of observations.

In principle, the equation with the lowest Mallow’s Cp value will be the optimal one. However, the residual sum of squares always decreases as dependent variables are added to the equation. Consequently, we will always select the equation with the highest number of indices if we aim for the minimum residual sum values. 

However, we must always bear in mind that the predictions made from a linear regression function are not reliable if there is not a high degree of linear correlation between the independent and dependent variables. In addition, its statistical quality must be analyzed and the higher it is, the more reliable and accurate the prediction will be. 

It is also recommended that the number of independent variables chosen be well below the number of observations, to avoid possible fortuitous correlations, so that the optimal relationship between them is the one that achieves a better prediction with the minimum number of descriptors possible, in order to avoid random variation as much as possible [55]. Randomness tests were also carried out to identify possible fortuitous regressions. To do this, the property value of each compound is randomly permuted and linearly correlated with the selected independent variables. This process is repeated as many times as necessary. The result of the randomness test is expressed by graphically representing the correlation coefficient (*r*^2^) versus the prediction coefficient (*r*^2^*_cv_*). 

For all those reasons, the number of observations (size of the set), the effect of the different independent variables and the degree of correlation between them will influence the selection of RFs under the criterion of this algorithm. 

## 5. Conclusions

The increase of resistant *Enterobacteriaceae* bacteria such as *E. aerogenes* and *P. mirabilis* is one of the major challenges that have appeared in recent years in infectious disease treatments. Therefore, the urge to find new compounds with antibacterial activity has not ceased to grow. It must also be noted that the pharmacokinetic profile of a compound is essential in determining the clinical outcome of an antibiotic treatment.

We have designed and developed three statistically significant, descriptor-independent regression functions with a high predictive capacity, which will aid us obtain theoretical values for the pharmacokinetic and microbiological properties selected. The results of the predictions obtained are supported by internal validation studies performed on all selected properties, as well as by all the statistical parameters, which can be classified as very satisfactory in all cases.

These equations, used as predictive models or as filters, can be extremely useful to find new molecules with antibacterial activity and even for their use in drug repositioning, where we find thousands of candidate compounds, which will have most certainly endured toxicological assays, to be screened in a short time for the search of safe antibacterial compounds.

We can conclude that the prediction functions obtained confirm molecular topology as a useful and efficient tool in the prediction of pharmacokinetic and microbiological properties. 

## Figures and Tables

**Table 1:** Prediction results obtained for the CL property (mL/min).

Compound	^6^ *χ_ch_*	*S_>N-_*	*NI*_2_	ExpCL ^a^	CalcCL ^b^	Res ^c^
Moxifloxacin	0.1741	3.803	3.319	14.8	88.2486	−73.4486
Amifloxacin	0.1842	5.5294	3.262	31.0	65.715	−34.715
Garenoxacin	0.169	1.6095	3.408	86.1	85.4983	0.6017
Pefloxacin	0.1842	5.8196	3.262	106.716	33.9436	72.7724
Trovafloxacin	0.2245	2.731	3.392	106.8132	93.5342	36.2154
Fleroxacin	0.1741	4.5829	3.265	127.126	145.2989	23.0078
Levofloxacin	0.2296	5.8165	3.219	198.0	220.1923	38.148
Temafloxacin	0.2523	2.9448	3.352	206.6	220.0219	38.5829
Sitafloxacin	0.0907	3.1603	3.261	263.0	178.4345	42.066
Clinafloxacin	0.0907	3.4961	3.146	368.857	445.0039	−76.1469
Enoxacin	0.203	3.3811	3.166	562.875	584.1476	−21.2727
Ciprofloxacin	0.203	3.7812	3.17	617.945	529.7932	88.1518

^a^ Experimental value of the CL property for each compound. ^b^ Calculated value of the CL property for each compound. ^c^ Residual value of RF_CL_ for each compound.

**Table 2:** Prediction results obtained for the MIC50Ea property (μg/mL).

Compound	*S* *_-CH2_*	*S* *_aCa_*	*S_-NH-_*	ExpMIC50Ea ^a^	CalcMIC50Ea ^b^	Res ^c^
Clinafloxacin	3.5533	0.1483	0	0.019	−0.0161	0.0351
Ciprofloxacin	4.8443	0.7434	3.2252	0.0351	0.0495	−0.0145
Tosufloxacin	1.5749	−2.6064	0	0.045	0.0384	0.0066
Gemifloxacin	2.7854	−0.3502	0	0.0465	0.0442	0.0023
Trovafloxacin	1.0072	−3.2996	0	0.048	0.0607	−0.0127
Levofloxacin	3.2033	0.6381	0	0.06	0.0606	−0.0006
Sparfloxacin	2.2119	−3.1814	3.3	0.084	0.1085	−0.0245
Fleroxacin	0.7962	−2.8908	0	0.09	0.1147	−0.0247
Norfloxacin	3.4104	0.6027	3.2081	0.1633	0.2153	−0.052
Enoxacin	3.1429	−0.1195	3.1803	0.2083	0.1971	0.0112
Amifloxacin	3.0361	0.3878	2.8144	0.25	0.2242	0.0258
Lomefloxacin	1.7121	−2.1985	3.2023	0.29	0.2318	0.0582
WIN-57273	1.8278	1.6636	0	0.29	0.3001	−0.0101

^a^ Experimental value of the MIC50Ea property for each compound. ^b^ Calculated value of the MIC50Ea property for each compound. ^c^ Residual value of RF_MIC50Ea_ for each compound.

**Table 3:** Prediction results obtained for the MIC50Pm property (μg/mL).

Compound	^9^ *χ^V^_ch_*	*S* *_-CH_*_3_	*S_>N-_*	ExpMIC50Pm ^a^	CalcMIC50Pm ^b^	Res ^c^
Clinafloxacin	0	0	3.4961	0.0225	0.0652	−0.0427
Sitafloxacin	0	0	3.1603	0.03	0.095	−0.065
Ciprofloxacin	0	0	3.7812	0.0426	0.0398	0.0028
Levofloxacin	0	3.8877	5.8165	0.06	0.1116	−0.0516
Flerofloxacin	0	1.8996	4.5829	0.12	0.092	0.028
Tosufloxacin	0	0	2.8277	0.12	0.1246	−0.0046
Gemifloxacin	0	1.437	3.3757	0.165	0.1693	−0.0043
Gatifloxacin	0	3.4792	3.6897	0.198	0.2741	−0.0761
Trovafloxacin	0	0	2.731	0.199	0.1332	0.0658
Amifloxacin	0	3.6197	5.5294	0.25	0.1197	0.1303
Enoxacin	0	1.8106	3.3811	0.25	0.1931	0.0569
Sparfloxacin	0	3.8376	3.0307	0.25	0.356	−0.1060
Lomefloxacin	0	3.6201	2.9493	0.29	0.3491	−0.0591
Garenoxacin	0.0049	2.0568	1.6095	0.5	0.5022	−0.0022
Moxifloxacin	0.0132	1.4781	3.803	0.5	0.4992	0.0008
WIN-57273	0	3.684	1.7957	0.583	0.4559	0.1271

^a^ Experimental value of the MIC50Pm property for each compound. ^b^ Calculated value of the MIC50Pm property for each compound. ^c^ Residual value of RF_MIC50Pm_ for each compound.

## Data Availability

This study did not report any data.

## Supplementary Materials

The following are available online at https://www.mdpi.com/article/10.3390/ph15020122/s1. Supplementary Section S1: Internal validation results for each of the selected equations; Supplementary Section S2: Compounds used in the realization of the equations: molecule name/code, IUPAC name & structure and bibliographic references about activity for each compound; Supplementary Section S3: Microsoft Excel file containing all the significant and non-redundant topological indices for the selected compounds; Supplementary Section S4: Symbols and Definitions of Topological Indices used with DESMOL13 and MOLCONN-Z programs.
