# Supplementary material for: New Pharmacokinetic and Microbiological Prediction Equations to Be Used as Models for the Search of Antibacterial Drugs
Source: Pharmaceuticals (Basel). 2022 Jan 20;15(2):122. doi: 10.3390/ph15020122 (PMC8879282; doi:10.3390/ph15020122)
Supplement: Supplementary file 1 [file pharmaceuticals-15-00122-s001.zip › Supplementary Section S1.pdf]

Article

New Pharmacokinetic and Microbiological Prediction Equations to Be Used as Models for the Search of Antibacterial Drugs

Jose I. Bueso-Bordils [1,*], Gerardo M. Antón-Fos [1], Antonio Falcó [2], Maria J. Duart [1], Rafael Martín-Algarra [1] and Pedro A. Alemán-López [1]

[1] Departamento de Farmacia, Universidad Cardenal Herrera-CEU, CEU Universities C/Ramón y Cajal s/n, Alfara del Patriarca, 46115 Valencia, Spain; ganton@uchceu.es (G.M.A.-F.); mduart@uchceu.es (M.J.D.); rmartin@uchceu.es (R.M.-A.); paleman@uchceu.es (P.A.A.-L.)

[2] ESI International Chair@CEU-UCH, Departamento de Matemáticas, Física y Ciencias Tecnológicas, Universidad Cardenal Herrera-CEU, CEU Universities San Bartolomé 55, Alfara del Patriarca, 46115 Valencia, Spain; afalco@uchceu.es

* Correspondence: jose.bueso@uchceu.es; Tel.: +34-96-136-9000

Supplementary Section S1: Internal validation results for each of the selected equations

Table S1. Standard error (SE) and significance (p) of the indices used in the equation RF_{CL}.

Index	SE	p
$^{6}\chi_{ch}$	441.367	0.007
$S_{>N-}$	17.6114	0.000
NI_2	278.522	0.000

Table S2. Correlations of the estimates of the regression coefficients of the indices used in the equation RF_{CL}.

Index	$^{6}\chi_{ch}$	$S_{>N-}$	NI_2
$^{6}\chi_{ch}$	1.000		
$S_{>N-}$	-0.316	1.000	
NI_2	-0.408	0.510	1.000

Figure S1. Residual values (res) versus cross-validated residual values (res(cv)) in the equation RF_{CL}.

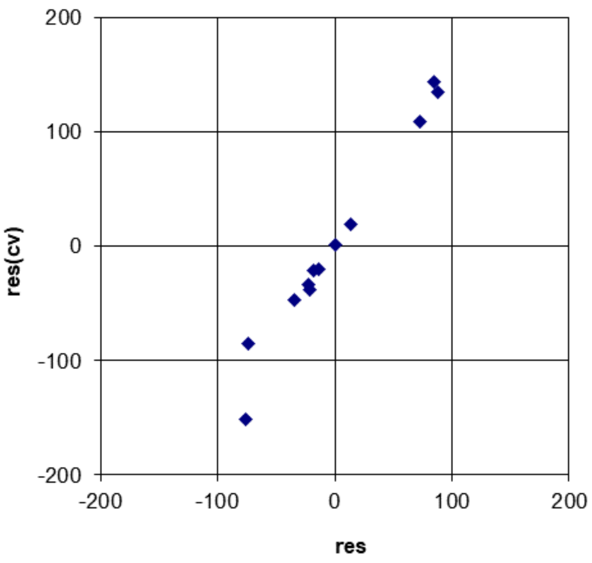

Figure S2. Correlation coefficient (r2) versus prediction coefficient (r2(vc)) in the equation RF_{CL}.

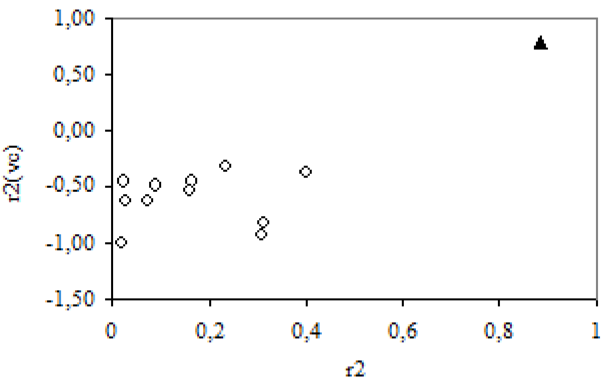

Table S3. Standard error (SE) and significance (p) of the indices used in the equation $RF_{MIC50Ea}$.

Index	SE	p
S_{-CH2-}	0.0139545	0.000
$S_{>N-}$	0.00827706	0.000
S_{-NH-}	0.00688546	0.000

Table S4. Correlations of the estimates of the regression coefficients of the indices used in the equation $RF_{MIC50Ea}$.

Index	S_{-CH2-}	S_{aCa}	S_{-NH-}
S_{-CH2-}	1.000		
S_{aCa}	-0.752	1.000	
S_{-NH-}	-0.531	0.355	1.000

Figure S3. Residual values (res) versus cross-validated residual values (res(cv)) in the equation $RF_{MIC50Ea}$.

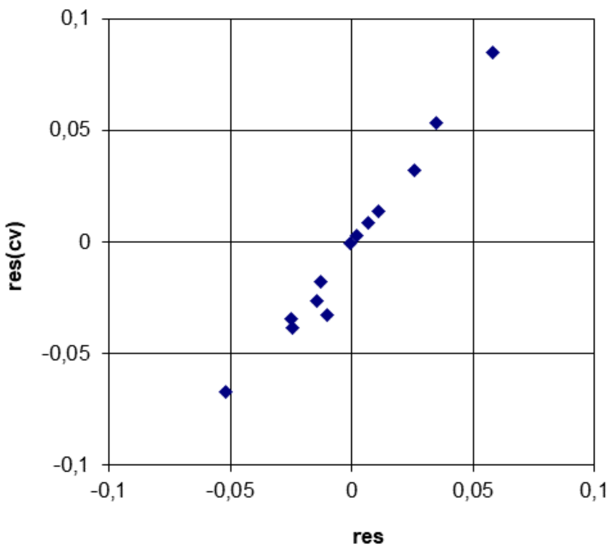

Figure S4. Correlation coefficient (r2) versus prediction coefficient (r2(vc)) in the equation $RF_{MIC50Ea}$.

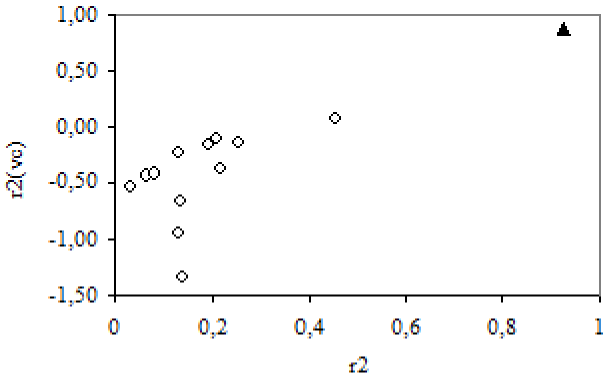

Table S5. Standard error (SE) and significance (p) of the indices used in the equation $RF_{MIC50Pm}$.

Index.	SE	p
$^9\chi^V_{ch}$	5.78657	0.000
$S_{>N-}$	0.0128396	0.000
S_{-NH-}	0.0181043	0.000

Table S6. Correlations of the estimates of the regression coefficients of the indices used in the equation $RF_{MIC50Pm}$.

Index.	$^9\chi^V_{ch}$	S_{-CH3}	$S_{>N-}$
$^9\chi^V_{ch}$	1.000		
S_{-CH3}	0.047	1.000	
$S_{>N-}$	0.069	-0.220	1.000

Figure S5. Residual values (res) versus cross-validated residual values (res(cv)) in the equation $RF_{MIC50Pm}$.

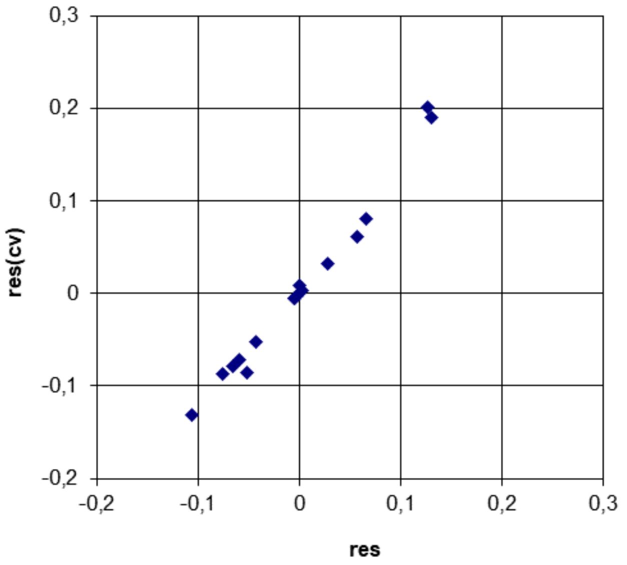

Figure S6. Correlation coefficient (r2) versus prediction coefficient (r2(vc)) in the equation $RF_{MIC50Pm}$.

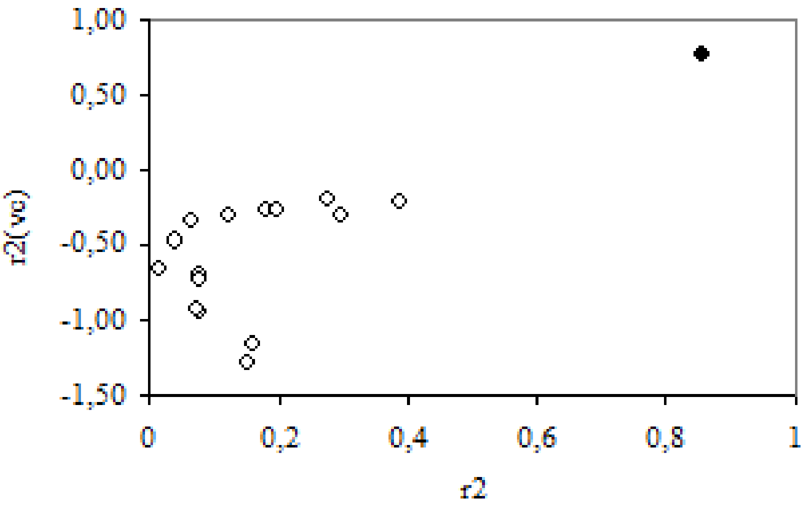
1,00
0,50
0,00
-0,50
-1,00
-1,50
r2(vc)
0
0,2
0,4
0,6
0,8
1
r2
