# Supplementary material for: New Pharmacokinetic and Microbiological Prediction Equations to Be Used as Models for the Search of Antibacterial Drugs
Source: Pharmaceuticals (Basel). 2022 Jan 20;15(2):122. doi: 10.3390/ph15020122 (PMC8879282; doi:10.3390/ph15020122)
Supplement: Supplementary file 1 [file pharmaceuticals-15-00122-s001.zip › Supplementary Section S2.pdf]

Article

New Pharmacokinetic and Microbiological Prediction Equations to Be Used as Models for the Search of Antibacterial Drugs

Jose I. Bueso-Bordils [1,*], Gerardo M. Antón-Fos [1], Antonio Falcó [2], Maria J. Duart [1], Rafael Martín-Algarra [1] and Pedro A. Alemán-López [1]

[1] Departamento de Farmacia, Universidad Cardenal Herrera-CEU, CEU Universities C/Ramón y Cajal s/n, Alfara del Patriarca, 46115 Valencia, Spain; ganton@uchceu.es (G.M.A.-F.); mduart@uchceu.es (M.J.D.); rmartin@uchceu.es (R.M.-A.); paleman@uchceu.es (P.A.A.-L.)

[2] ESI International Chair@CEU-UCH, Departamento de Matemáticas, Física y Ciencias Tecnológicas, Universidad Cardenal Herrera-CEU, CEU Universities San Bartolomé 55, Alfara del Patriarca, 46115 Valencia, Spain; afalco@uchceu.es

* Correspondence: jose.bueso@uchceu.es; Tel.: +34-96-136-9000

Supplementary Section S2: Compounds used in the realization of the equations: molecule name/code, IUPAC name & structure and bibliographic references about activity for each compound.

Paper name/code	IUPAC name & structure	References
A-80556	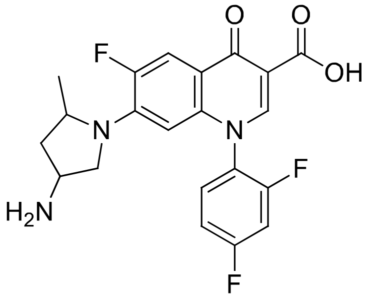 7-(4-amino-2-methylpyrrolidin-1-yl)-1-(2,4-difluorophenyl)-6-fluoro-4-oxo-1,4-dihydroquinoline-3-carboxylic acid	Clement, J.J.; Tanaka, S.K.; Alder, J.; Vojtko, C.; Beyer, J.; Hensey, D.; Ramer, N.; McDaniel, D.; Chu, D.T. *In vitro* and *in vivo* evaluations of A-80556, a new fluoroquinolone. *Antimicrob. Agents Chemother.*, **1994**, *38*(5), 1071-8.
Amifloxacin	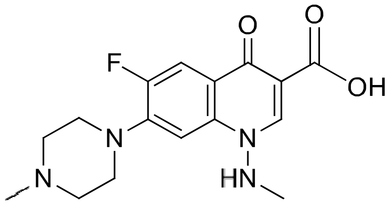 6-fluoro-1-(methylamino)-7-(4-methylpiperazin-1-yl)-4-oxo-1,4-dihydroquinoline-3-carboxylic acid	Stroshane, R.M.; Silverman, M.H.; Sauerschell, R.; Brown, R.R.; Boddy, A.W.; Cook, J.A. Preliminary study of the pharmacokinetics of oral amifloxacin in elderly subjects. *Antimicrob. Agents Chemother.*, **1990**, *34*(5), 751-4.<br>Lode, H. Pharmacokinetics and clinical results of parenterally administered new quinolones in humans. *Rev. Infect. Dis.*, **1989**, *11* (S5), S996-1004.<br>Sedlock, D.M.; Dobson, R.A.; Deuel, D.M.; Lesher, G.Y.; Rake, J.B. *In vitro* and *in vivo* activities of a new quinolone, WIN 57273, possessing potent activity against gram-positive bacteria. *Antimicrob. Agents Chemother.K,* **1990**, *34*(4), 568-75.<br>Venezia, R.A.; Prymas, L.A.; Shayegani, A.; Yocum, D.M. *In vitro* activities of amifloxacin and two of its metabolites. *Antimicrob. Agents Chemother.*,**1989**, *33*(5), 762-6.
Balofloxacin	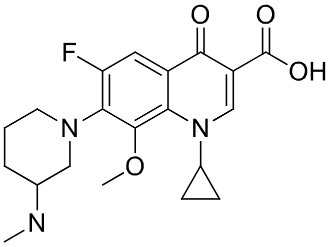 1-cyclopropyl-6-fluoro-8-methoxy-7-(3-(methylamino)piperidin-1-yl)-4-oxo-1,4-dihydroquinoline-3-carboxylic acid	Nakashima, M.; Uematsu, T.; Fukuchi, M.; Nakano, M.; Kosuge, K. Clinical phase I study of balofloxacin I. Single oral administration. *Jpn. J. Antimicrob. Chemother.*, **1995**, *43*(S5), 115-40.<br>Ito, T.; Otsuki, M.; Nishino, T. *In vitro* antibacterial activity of Q-35, a new fluoroquinolone. *Antimicrob. Agents Chemother.*, **1992**, *36*(8), 1708-1714.

BAY Y3118	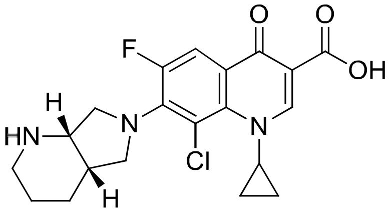<br>8-chloro-1-cyclopropyl-6-fluoro-7-((4aS,7aS)-hexahydro-1H-pyrrolo[3,4-b]pyridin-6(2H)-yl)-4-oxo-1,4- dihydroquinoline-3-carboxylic acid	Bongaerts, G.P.A.; Hoogkamp, J.A.A. *In vitro* activities of BAY Y3118, ciprofloxacin, ofloxacin, and fleroxacin against gram-positive and gram-negative pathogens from respiratory tract and soft tissue infections. *Antimicrob. Agents Chemother.*, **1993**, *37*(9), 2017-9.<br>Borobio, M.V.; Conejo, M.; Ramirez, E.; Suarez, A.I.; Perea, E.J. Comparative activities of eight quinolones against members of the Bacteroides fragilis group. *Antimicrob. Agents Chemother.*, **1994**, *38*(6), 1442-5.<br>Wexler, H.M.; Molitoris, E.; Finegold, S.M. *In vitro* activity of BayY3118 against anaerobic bacteria. *Antimicrob. Agents Chemother.*, **1993**, *37*(11), 2509-13.
Cetefloxacin	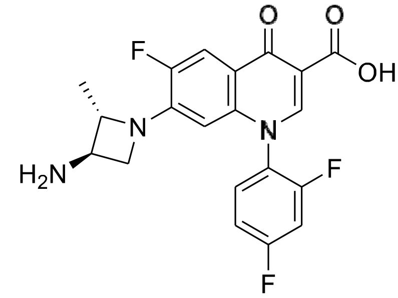<br>7-((2S,3R)-3-amino-2-methylazetidin-1-yl)-1-(2,4-difluorophenyl)-6-fluoro-4-oxo-1,4-dihydroquinoline-3-carboxylic acid	Borobio, M.V.; Conejo, M.; Ramirez, E.; Suarez, A.I.; Perea, E.J. Comparative activities of eight quinolones against members of the Bacteroides fragilis group. *Antimicrob. Agents Chemother.*, **1994**, *38*(6), 1442-5.<br>Guinea, J.; Robert, M.; Gargallo-Viola, D.; Xicota, M.A.; Garcia, J.; Tudela, E.; Esteve, M.; Coll, R.; Pares, M.; Roser, R. *In vitro* and *in vivo* antibacterial activities of E-4868, a new fluoroquinolone with a 7-azetidin ring substituent. *Antimicrob. Agents Chemother.*, **1993**, *37*(4), 868-74.
CFC-222	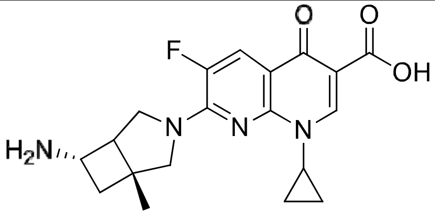<br>7-((1R,6S)-6-amino-1-methyl-3- azabicyclo[3.2.0]heptan-3-yl)-1- cyclopropyl-6-fluoro-4-oxo-1,4-dihydro- 1,8-naphthyridine-3-carboxylic acid	Kim, J.H.; Choi, K.H.; Kim, J.W.; Lee, J.H.; Choi, E.C.; Kim, B.K. *In-vitro* and *in-vivo* antibacterial activity of CFC-222, a new fluoroquinolone. *J. Antimicrob. Chemother.*, **1998**, *41*(2), 223-9.<br>Kim, J.H.; Kang, J.A.; Lee, Y.; Lee, K.H.; Lee, J.H.; Choi, E.C.; Kim, B.K. Susceptibility of penicillin-susceptible and -resistant pneumococci to CFC-222, a new fluoroquinolone. *J. Antimicrob. Chemother.*, **1998**, *42*(4), 527-30.
Ciprofloxacin	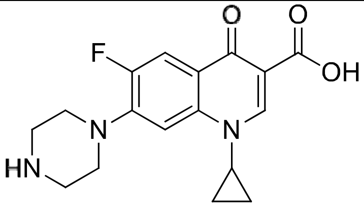	Meagher, A.K.; Forrest, A.; Dalhoff, A.; Stass, H.; Schentag, J.J. Novel pharmacokinetic-pharmacodynamic model for prediction of outcomes with an extended-release formulation of ciprofloxacin. *Antimicrob. Agents*
	1-cyclopropyl-6-fluoro-4-oxo-7-(piperazin-1-yl)-1,4-dihydroquinoline-3-carboxylic	*Chemother.*, **2004**, *48*(6), 2061-8.<br>Wagenlehner, F.M.; Wydra, S.; Onda,

	acid	H.; Kinzig-Schippers, M.; Sörgel, F.; Naber, KG. Concentrations in plasma, urinary excretion, and bactericidal activity of linezolid (600 milligrams) versus those of ciprofloxacin (500 milligrams) in healthy volunteers receiving a single oral dose. *Antimicrob. Agents Chemother.*, **2003**, *47*(12), 3789-94.<br>Kamberi, M.; Tsutsumi, K.; Kotegawa, T.; Kawano, K.; Nakamura, K.; Niki, Y.; Nakano, S. Influences of urinary pH on ciprofloxacin pharmacokinetics in humans and antimicrobial activity *in vitro* versus those of sparfloxacin. *Antimicrob. Agents Chemother.*, **1999**, *43*(3), 525-9.<br>Lettieri, J.T.; Rogge, M.C.; Kaiser, L.; Echols, R.M.; Heller, A.H. Pharmacokinetic profiles of ciprofloxacin after single intravenous and oral doses. *Antimicrob Agents Chemother.*, **1992**, *36*(5), 993-6.<br>Barriere, S.L.; Catlin, D.H.; Orlando, P.L.; Noe, A; Frost, R.W. Alteration in the pharmacokinetic disposition of ciprofloxacin by simultaneous administration of azlocillin. *Antimicrob. Agents Chemother.*, **1990**, *34*(5), 823-6.<br>Yuk, J.H.; Nightingale, C.H.; Sweeney, K.R.; Quintiliani, R.; Lettieri, J.T.; Frost, R.W. Relative bioavailability in healthy volunteers of ciprofloxacin administered through a nasogastric tube with and without enteral feeding. *Antimicrob. Agents Chemother.*, **1989**, *33*(7), 1118-20.<br>Dudley, M.N.; Ericson, J.; Zinner, S.H. Effect of dose on serum pharmacokinetics of intravenous ciprofloxacin with identification and characterization of extravascular compartments using noncompartmental and compartmental pharmacokinetic models. *Antimicrob. Agents Chemother.*, **1987**, *31*(11), 1782-6.

		Plaisance, K.I.; Drusano, G.L.; Forrest, A.; Bustamante, C.I.; Standiford, H.C. Effect of dose size on bioavailability of ciprofloxacin. *Antimicrob. Agents Chemother.*, **1987**, *31*(6), 956-8.<br>Drusano, G.L.; Standiford, H.C.; Plaisance, K.; Forrest, A.; Leslie, J.; Caldwell, J. Absolute oral bioavailability of ciprofloxacin. *Antimicrob. Agents Chemother.*, **1986**, *30*(3), 444-6.<br>LeBel, M.; Bergeron, M.G.; Vallée, F.; Fiset, C.; Chassé, G.; Bigonesse, P.; Rivard, G. Pharmacokinetics and pharmacodynamics of ciprofloxacin in cystic fibrosis patients. *Antimicrob. Agents Chemother.*, **1986**, *30*(2), 260-6.<br>Lode, H.; Höffken, G.; Boeckk, M.; Deppermann, N.; Borner, K.; Koeppe, P. Quinolone pharmacokinetics and metabolism. *J. Antimicrob. Chemother.*, **1990**, 26(SB), 41-9.<br>Cormican, M.G.; Jones, R.N. Antimicrobial activity and spectrum of LB20304, a novel fluoronaphthyridone. *Antimicrob. Agents Chemother.*, **1997**, *41*(1), 204-11.<br>Tanaka, M.; Yamazaki, E.; Chiba, M.; Yoshihara, K.; Akasaka, T.; Takemura, M.; Sato, K. *In vitro* antibacterial activities of DQ-113, a potent quinolone, against clinical isolates. *Antimicrob. Agents Chemother.*, **2002**, *46*(3), 904-8.<br>Brueggemann, A.B.; Kugler, K.C.; Doern, G.V. *In vitro* activity of BAY 12-8039, a novel 8-methoxyquinolone, compared to activities of six fluoroquinolones against *Streptococcus pneumoniae*, *Haemophilus influenzae*, and *Moraxella catarrhalis*. *Antimicrob. Agents Chemother.*, **1997**, *41*(7), 1594-7.<br>Montanari, M.P.; Mingoia, M.; Varaldo, P.E. *In vitro* antibacterial activities of AF 3013, the active metabolite of prulifloxacin, against nosocomial

		and community Italian isolates. *Antimicrob. Agents Chemother.*, **2001**, *45*(12), 3616-22.<br>Yun, H.J.; Min, Y.H.; Lim, J.A.; Kang, J.W.; Kim, S.Y.; Kim, M.J.; Jeong, J.H.; Choi, Y.J.; Kwon, H.J.; Jung, Y.H.; Shim, M.J.; Choi, EC. *In vitro* and *in vivo* antibacterial activities of DW286, a new fluoronaphthyridone antibiotic. *Antimicrob. Agents Chemother.*, **2002**, *46*(9), 3071-4.<br>Hoellman, D.B.; Kelly, L.M.; Jacobs, M.R.; Appelbaum, P.C. Comparative antianaerobic activity of BMS 284756. *Antimicrob. Agents Chemother.*, **2001**, *45*(2), 589-92.<br>Gargallo-Viola, D.; Ferrer, S.; Tudela, E.; Robert, M.; Coll, R.; Roser, R, Guinea J. Antibacterial activities and pharmacokinetics of E-4767 and E-5065, two new 8-chlorofluoroquinolones with a 7-azetidin ring substituent. *Antimicrob. Agents Chemother.*, **2001**, *45*(11), 3113-21.<br>Neu, H.C.; Chin, N.X. *In vitro* activity of the new fluoroquinolone CP-99,219. *Antimicrob. Agents Chemother.*, **1994**, *38*(11), 2615-22.<br>Neu, H.C.; Fang, W.; Gu, J.W.; Chin, N.X. *In vitro* activity of OPC-17116. *Antimicrob. Agents Chemother.*, **1992**, *36*(6), 1310-5.<br>Coll, R.; Gargallo-Viola, D.; Tudela, E.; Xicota, M.A.; Llovera, S.; Guinea, J. Antibacterial activity and pharmacokinetics of four new 7- azetidinyl fluoroquinolones. *Antimicrob. Agents Chemother.*, **1996**, *40*(1), 274-7.<br>Almer, L.S.; Hoffrage, J.B.; Keller, E.L.; Flamm, R.K.; Shortridge, V.D. *In vitro* and bactericidal activities of ABT- 492, a novel fluoroquinolone, against Gram-positive and Gram-negative organisms. *Antimicrob. Agents Chemother.*, **2004**, *48*(7), 2771-7.<br>Nilius, A.M.; Shen, L.L.; Hensey-Rudloff, D.; Almer, L.S.; Beyer, J.M.;

		Balli, D.J.; Cai, Y.; Flamm, R.K. *In vitro* antibacterial potency and spectrum of ABT-492, a new fluoroquinolone. *Antimicrob. Agents Chemother.*, **2003**, *47*(10), 3260-9.<br>Yong, D.E.; Cheong, H.J.; Kim, Y.S.; Park, Y.J.; Kim, W.J.; Woo, J.H.; Lee, K.W.; Kang, M.W.; Choo, Y.S. *In vitro* activity of gemifloxacin against recent clinical isolates of bacteria in Korea. *J. Korean Med. Sci.*, **2002**, *17*(6), 737-42.<br>Fung-Tomc, J.; Minassian, B.; Kolek, B.; Washo, T.; Huczko, E.; Bonner, D. *In vitro* antibacterial spectrum of a new broad-spectrum 8-methoxy fluoroquinolone, gatifloxacin. *J. Antimicrob. Chemother.*, **2000**, *45*(4), 437-46.<br>Schaumann, R.; Ackermann, G.; Pless, B.; Claros, M.C.; Rodloff, A.C. *In vitro* activities of gatifloxacin, two other quinolones, and five nonquinolone antimicrobials against obligately anaerobic bacteria. *Antimicrob. Agents Chemother.*, **1999**, *43*(11), 2783-6.<br>Jones, R.N.; Croco, M.A.; Pfaller, M.A.; Beach, M.L.; Kugler, K.C. Antimicrobial activity evaluations of gatifloxacin, a new fluoroquinolone: contemporary pathogen results from a global antimicrobial resistance surveillance program (SENTRY, 1997). *Clin. Microbiol. Infect.*, **1999**, *5*(9), 540-6.<br>Kim, J.H.; Choi, K.H.; Kim, J.W.; Lee, J.H.; Choi, E.C.; Kim, B.K. *In-vitro* and *in-vivo* antibacterial activity of CFC-222, a new fluoroquinolone. *J. Antimicrob. Chemother.*, **1998**, *41*(2), 223-9.<br>Felmingham, D.; Robbins, M.J.; Ingley, K.; Mathias, I.; Bhogal, H.; Leakey, A.; Ridgway, G.L.; Grüneberg, R.N. *In- vitro* activity of trovafloxacin, a new fluoroquinolone, against recent clinical isolates. *J. Antimicrob. Chemother.*, **1997**, *39*(SB), 43-9.<br>Cunha, B.A.; Qadri, S.M.; Ueno, Y.; Walters, E.A.; Domenico, P.

		Antibacterial activity of trovafloxacin against nosocomial Gram-positive and Gram-negative isolates. *J. Antimicrob. Chemother.*, **1997**, *39*(SB), 29-34.<br>Endtz, H.P.; Mouton, J.W.; den Hollander, J.G.; van den Braak, N.; Verbrugh, H.A. Comparative *in vitro* activities of trovafloxacin (CP-99,219) against 445 gram-positive isolates from patients with endocarditis and those with other bloodstream infections. *Antimicrob. Agents Chemother.*, **1997**, *41*(5), 1146-9.<br>Choi, K.H.; Hong, J.S.; Kim, S.K.; Lee, D.K.; Yoon, S.J.; Choi, E.C. *In-vitro* and *in-vivo* activities of DW-116, a new fluoroquinolone. *J. Antimicrob. Chemother.*, **1997**, *39*(4), 509-14.<br>Clement, J.J.; Tanaka, S.K.; Alder, J.; Vojtko, C.; Beyer, J.; Hensey, D.; Ramer, N.; McDaniel, D.; Chu, D.T. *In vitro* and *in vivo* evaluations of A-80556, a new fluoroquinolone. *Antimicrob. Agents Chemother.*, **1994**, *38*(5), 1071-8.<br>Guinea, J.; Robert, M.; Gargallo-Viola, D.; Xicota, M.A.; Garcia, J.; Tudela, E.; Esteve, M.; Coll, R.; Pares, M.; Roser, R. *In vitro* and *in vivo* antibacterial activities of E-4868, a new fluoroquinolone with a 7-azetidin ring substituent. *Antimicrob. Agents Chemother.*, **1993**, *37*(4), 868-74.<br>Chin, N.X.; Gu, J.W.; Yu, K.W.; Zhang, Y.X.; Neu, H.C. *In vitro* activity of sparfloxacin. *Antimicrob. Agents Chemother.*, **1991**, *35*(3), 567-71.<br>Eliopoulos, G.M.; Klimm, K.; Rice, L.B.; Ferraro, M.J.; Moellering, R.C. Jr. Comparative *in vitro* activity of WIN 57273, a new fluoroquinolone antimicrobial agent. *Antimicrob. Agents Chemother.*, **1990**, *34*(6), 1154-9.<br>Jones, R.N.; Barry, A.L. *In vitro* evaluation of WIN 57273, a new broad-spectrum fluoroquinolone.

	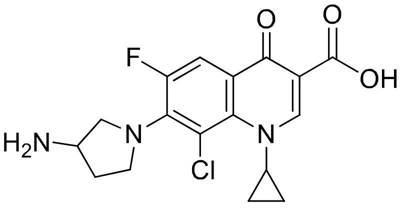	*Antimicrob. Agents Chemother.*, **1990**, *34*(2), 306-13.<br>Barry, A.L.; Fuchs, P.C.; Brown, S.D. *In vitro* activities of three nonfluorinated quinolones against representative bacterial isolates. *Antimicrob. Agents Chemother.*, **2001**, *45*(6), 1923-7.<br>Borobio, M.V.; Conejo, M.; Ramirez, E.; Suarez, A.I.; Perea, E.J. Comparative activities of eight quinolones against members of the Bacteroides fragilis group. *Antimicrob. Agents Chemother.*, **1994**, *38*(6), 1442-5.<br>Bongaerts, G.P.A.; Hoogkamp, J.A.A. *In vitro* activities of BAY Y3118, ciprofloxacin, ofloxacin, and fleroxacin against gram-positive and gram-negative pathogens from respiratory tract and soft tissue infections. *Antimicrob. Agents Chemother.*, **1993**, *37*(9), 2017-9.<br>Goldstein, E.J.; Citron, D.M. Comparative activity of ciprofloxacin, ofloxacin, sparfloxacin, temafloxacin, CI-960, CI-990, and WIN 57273 against anaerobic bacteria. *Antimicrob. Agents Chemother.*, **1992**, *36*(5), 1158-62.<br>Sedlock, D.M.; Dobson, R.A.; Deuel, D.M.; Lesher, G.Y.; Rake, J.B. *In vitro* and *in vivo* activities of a new quinolone, WIN 57273, possessing potent activity against gram-positive bacteria. *Antimicrob. Agents Chemother.K,* **1990**, *34*(4), 568-75.<br>Venezia, R.A.; Prymas, L.A.; Shayegani, A.; Yocum, D.M. *In vitro* activities of amifloxacin and two of its metabolites. *Antimicrob. Agents Chemother.*, **1989**, *33*(5), 762-6.<br>Rolston, K.V.; Ho, D.H.; LeBlanc, B.; Bodey, G.P. *In vitro* evaluation of S-25930 and S-25932, two new quinolones, against aerobic gram-negative isolates from cancer patients. *Antimicrob. Agents Chemother.*, **1987**, *31*(1), 102-3.<br>García-Rodríguez, J.A.; García Sánchez, J.E.; Muñoz Bellido, J.L.; Trujillano, I. *In vitro* activities of irloxacin and E-3846, two new quinolones. *Antimicrob. Agents Chemother.*, **1990**, *34*(6), 1262-7.

		Otani, T.; Tanaka, M.; Ito, E.; Kurosaka, Y.; Murakami, Y.; Onodera,

		K.; Akasaka, T.; Sato, K. *In vitro* and *in vivo* antibacterial activities of DK-507k, a novel fluoroquinolone. *Antimicrob. Agents Chemother.*, **2003**, *47*(12), 3750-9.<br>Roychoudhury, S.; Twinem, T.L.; Makin, K.M.; McIntosh, E.J.; Ledoussal, B.; Catrenich, C.E.<br>Activity of non-fluorinated quinolones (NFQs) against quinolone-resistant *Escherichia coli* and *Streptococcus pneumoniae*.<br>*J. Antimicrob. Chemother.*, **2001**, *48*(1), 29-36.<br>Gooding, B.B.; Jones, R.N. *In vitro* antimicrobial activity of CP-99,219, a novel azabicyclo-naphthyridone.<br>*Antimicrob. Agents Chemother.*, **1993**, *37*(2), 349-53.
Clinafloxacin	7-(3-aminopyrrolidin-1-yl)-8-chloro-1-cyclopropyl-6-fluoro-4-oxo-1,4- dihydroquinoline-3-carboxylic acid	Wise, R.; Jones, S.; Das, I.; Andrews JM. Pharmacokinetics and inflammatory fluid penetration of clinafloxacin. *Antimicrob. Agents Chemother.*, **1998**, *42*(2), 428-30.<br>Randinitis, E.J.; Brofuehrer, J.I.; Eiseman, I.; Vassos, A.B. Pharmacokinetics of clinafloxacin after single and multiple doses.<br>*Antimicrob. Agents Chemother.*, **2001**, *45*(9), 2529-35.<br>Bron, N.J.; Dorr, M.B.; Mant, T.G.; Webb, C.L.; Vassos, A.B. The tolerance and pharmacokinetics of cli nafloxacin (CI-960) in<br>healthy subjects. *J. Antimicrob. Chemother.*, **1996**, *38*(6), 1023-9.<br>Biedenbach, D.J.; Sutton, L.D.; Jones, R.N. Antimicrobial activity of CS-940, a new trifluorinated quinolone.<br>*Antimicrob. Agents Chemother.*, **1995**, *39*(10), 2325-2330.<br>Snydman, D.R.; Jacobus, N.V.; McDermott, L.A.; Ruthazer, R.; Goldstein, E.; Finegold, S.; Harrell, L.; Hecht, D.W.; Jenkins, S.; Pierson, C.; Venezia, R.; Rihs, J; Gorbach, S.L. *In vitro* activities of newer quinolones against bacteroides group organisms.

	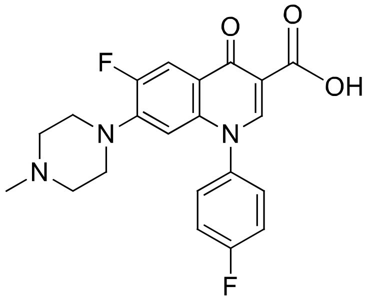	*Antimicrob. Agents Chemother.*, **2002**, *46*(10), 3276-9.<br>Milatovic, D.; Schmitz, F.J.; Brisse, S.; Verhoef, J.; Fluit, A.C. *In vitro* activities of sitafloxacin (DU-6859a) and six other fluoroquinolones against 8,796 clinical bacterial isolates. *Antimicrob. Agents Chemother.*, **2000**, *44*(4), 1102-7.<br>Roychoudhury, S.; Twinem, T.L.; Makin, K.M.; McIntosh, E.J.; Ledoussal, B.; Catrenich, C.E.<br>Activity of non-fluorinated quinolones (NFQs) against quinolone-resistant *Escherichia coli* and *Streptococcus pneumoniae*.<br>*J. Antimicrob. Chemother.*, **2001**, *48*(1), 29-36.<br>Brueggemann, A.B.; Kugler, K.C.; Doern, G.V.<br>*In vitro* activity of BAY 12-8039, a novel 8-methoxyquinolone, compared to activities of six fluoroquinolones against *Streptococcus pneumoniae*, *Haemophilus influenzae*, and *Moraxella catarrhalis*. *Antimicrob. Agents Chemother.*, **1997**, *41*(7), 1594-7.<br>Borobio, M.V.; Conejo, M.; Ramirez, E.; Suarez, A.I.; Perea, E.J. Comparative activities of eight quinolones against members of the Bacteroides fragilis group. *Antimicrob. Agents Chemother.*, **1994**, *38*(6), 1442-5.<br>Goldstein, E.J.; Citron, D.M. Comparative activity of ciprofloxacin, ofloxacin, sparfloxacin, temafloxacin, CI-960, CI-990, and WIN 57273 against anaerobic bacteria. *Antimicrob. Agents Chemother.*, **1992**, *36*(5), 1158-62.
CS-940	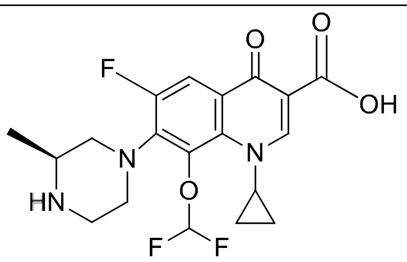<br>(S)-1-cyclopropyl-8-(difluoromethoxy)-6-fluoro-7-(3-methylpiperazin-1-yl)-4-oxo-1,4-dihydroquinoline-3-carboxylic acid	Biedenbach, D.J.; Sutton, L.D.; Jones, R.N. Antimicrobial activity of CS-940, a new trifluorinated quinolone. *Antimicrob. Agents Chemother.*, **1995**, *39*(10), 2325-2330.

Delafloxacin	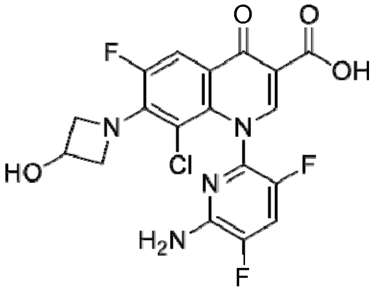<br>1-(6-amino-3,5-difluoropyridin-2-yl)-8-chloro-6-fluoro-7-(3-hydroxyazetidin-1-yl)-4-oxo-1,4-dihydroquinoline-3-carboxylic acid	Almer, L.S.; Hoffrage, J.B.; Keller, E.L.; Flamm, R.K.; Shortridge, V,D. *In vitro* and bactericidal activities of ABT- 492, a novel fluoroquinolone, against Gram-positive and Gram-negative organisms. *Antimicrob. Agents Chemother.*, **2004**, *48*(7), 2771-7.<br>Nilius, A.M.; Shen, L.L.; Hensey-Rudloff, D.; Almer, L.S.; Beyer, J.M.; Balli, D.J.; Cai, Y.; Flamm, R.K. *In vitro* antibacterial potency and spectrum of ABT-492, a new fluoroquinolone. *Antimicrob. Agents Chemother.*, **2003**, *47*(10), 3260-9.<br>Goldstein, E.J.; Citron, D.M.; Merriam, C.V.; Warren, Y.A.; Tyrrell, K.L.; Fernandez, H.T. *In vitro* activities of ABT-492, a new fluoroquinolone, against 155 aerobic and 171 anaerobic pathogens isolated from antral sinus puncture specimens from patients with sinusitis. *Antimicrob. Agents Chemother.*, **2003**, *47*(9), 3008-11.
Difloxacin	6-fluoro-1-(4-fluorophenyl)-7-(4-methylpiperazin-1-yl)-4-oxo-1,4-dihydroquinoline-3-carboxylic acid	McCaffrey, C.; Bertasso, A.; Pace, J.; Georgopapadakou, N.H. Quinolone accumulation in *Escherichia coli*, *Pseudomonas aeruginosa*, and *Staphylococcus aureus*. *Antimicrob. Agents Chemother.*, 1992, *36*(8), 1601-5.<br>Michéa-Hamzehpour, M.; Furet, Y.X.; Pechère, J.C. Role of protein D2 and lipopolysaccharide in diffusion of quinolones through the outer membrane of *Pseudomonas aeruginosa*. *Antimicrob. Agents Chemother.*, **1991**, *35*(10), 2091-7.<br>Chapman, J.S.; Georgopapadakou, N.H. Routes of quinolone permeation in *Escherichia coli*. *Antimicrob. Agents Chemother.*, **1988**, *32*(4), 438-42.<br>Granneman, G.R.; Snyder, K.M.; Shu, V.S. Difloxacin metabolism and pharmacokinetics in humans after single oral doses. *Antimicrob. Agents Chemother.*, **1986**, *30*(5), 689-93.<br>Stamm, J.M.; Hanson, C.W.; Chu, D.T.; Bailer, R.; Vojtko, C.; Fernandes, P.B. *In vitro* evaluation of A-56619 (difloxacin) and A-56620: new aryl-
		fluoroquinolones. *Antimicrob. Agents Chemother.*, **1986**, *29*(2), 193-200.

DK-507k	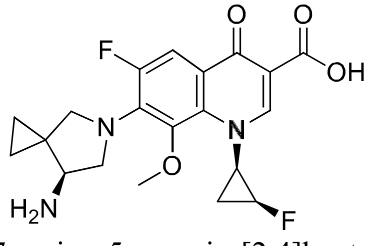 7-((S)-7-amino-5-azaspiro[2.4]heptan-5-yl)-6-fluoro-1-((1R,2S)-2-fluorocyclopropyl)-8-methoxy-4-oxo-1,4-dihydroquinoline-3-carboxylic acid	Otani, T.; Tanaka, M.; Ito, E.; Kurosaka, Y.; Murakami, Y.; Onodera, K.; Akasaka, T.; Sato, K. *In vitro* and *in vivo* antibacterial activities of DK-507k, a novel fluoroquinolone. *Antimicrob. Agents Chemother.*, **2003**, *47*(12), 3750-9.
DQ-113	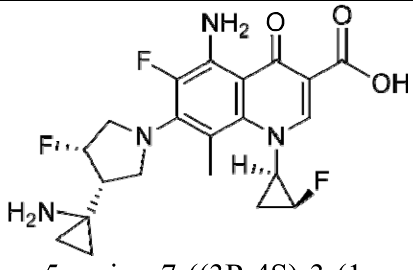 5-amino-7-((3R,4S)-3-(1-aminocyclopropyl)-4-fluoropyrrolidin-1-yl)-6-fluoro-1-((1R,2S)-2-fluorocyclopropyl)-8-methyl-4-oxo-1,4-dihydroquinoline-3-carboxylic acid	Tanaka, M.; Yamazaki, E.; Chiba, M.; Yoshihara, K.; Akasaka, T.; Takemura, M.; Sato, K. *In vitro* anti-bacterial activities of DQ-113, a potent quinolone, against clinical isolates. *Anti-microb. Agents Chemother.*, **2002**, *46*(3), 904-8.
DV-7751a	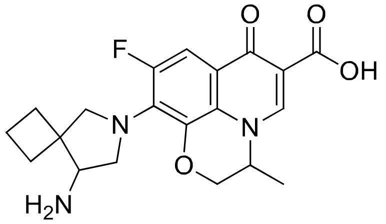 10-(8-amino-6-azaspiro[3.4]octan-6-yl)-9-fluoro-3-methyl-7-oxo-3,7-dihydro-2H-[1,4]oxazino[2,3,4-ij]quinoline-6-carboxylic acid	Tanaka, M.; Tamura, K.; Atarashi, S.; Kubo, Y.; Oliver, S.D.; Bentley, M.; Hakusui, H. Pharmacokinetics and toler-ance of a new fluoroquinolone antimicro-bial drug after single oral doses in healthy volunteers. *Xenobiotica*, **1995**, *25*(10), 1119-25.<br>Biedenbach, D.J.; Jones, R.N. *In vitro* evaluation of DV-7751a, a new fluoro-quinolone with an enhanced spectrum of activity against gram- positive aerobic organisms and<br>anaerobes. *Antimicrob. Agents Chemoth-er.*, **1995**, *39*(7), 1636-1643.
DW286	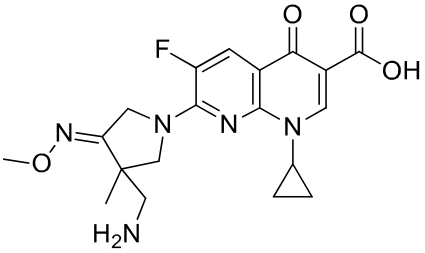 (E)-7-(3-(aminomethyl)-4-(methoxyimino)-3-methylpyrrolidin-1-yl)-1-cyclopropyl-6-fluoro-4-oxo-1,4-dihydro-1,8-	Yun, H.J.; Min, Y.H.; Lim, J.A.; Kang, J.W.; Kim, S.Y.; Kim, M.J.; Jeong, J.H.; Choi, Y.J.; Kwon, H.J.; Jung, Y.H.; Shim, M.J.; Choi, E.C. *In vitro* and *in vi-vo* antibacterial activities of DW286, a new fluoronaphthyridone antibiotic. *An-timicrob. Agents Chemother.*, **2002**, *46*(9), 3071-4.
	naphthyridine-3-carboxylic acid	

E-4767	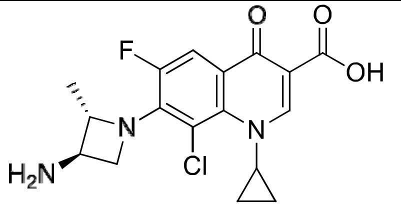<br>7-((2S,3R)-3-amino-2-methylazetidin-1-yl)-8-chloro-1-cyclopropyl-6-fluoro-4-oxo-1,4-dihydroquinoline-3-carboxylic acid	Gargallo-Viola, D.; Ferrer, S.; Tudela, E.; Robert, M.; Coll, R.; Roser, R.; Guinea, J. Antibacterial activities and pharmacokinetics of E-4767 and E-5065, two new 8-chlorofluoroquinolones with a 7-azetidin ring substituent. *Antimicrob. Agents Chemother.*, **2001**, *45*(11), 3113-21.
E-5065	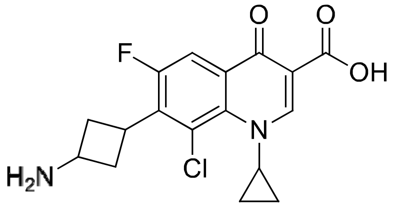<br>7-(3-aminocyclobutyl)-8-chloro-1-cyclopropyl-6-fluoro-4-oxo-1,4-dihydroquinoline-3-carboxylic acid	Gargallo-Viola, D.; Ferrer, S.; Tudela, E.; Robert, M.; Coll, R.; Roser, R.; Guinea, J. Antibacterial activities and pharmacokinetics of E-4767 and E-5065, two new 8-chlorofluoroquinolones with a 7-azetidin ring substituent. *Antimicrob. Agents Chemother.*, **2001**, *45*(11), 3113-21.<br>Borobio, M.V.; Conejo, M.; Ramirez, E.; Suarez, A.I.; Perea, E.J. Comparative activities of eight quinolones against members of the Bacteroides fragilis group. *Antimicrob. Agents Chemother.*, **1994**, *38*(6), 1442-5.<br>Wexler, H.M.; Molitoris, E.; Finegold, S.M. *In vitro* activity of BayY3118 against anaerobic bacteria. *Antimicrob. Agents Chemother.*, **1993**, *37*(11), 2509-13.
Enoxacin	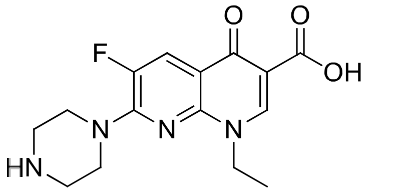<br>1-ethyl-6-fluoro-4-oxo-7-(piperazin-1-yl)-1,4-dihydro-1,8-naphthyridine-3-carboxylic acid	Wolfson, J.S.; Hooper, D.C. Fluoroquinolone antimicrobial agents. *Clin. Microbiol. Rev.*, **1989**, *2*(4), 378-424.<br>Lode, H.; Höffken, G.; Boeckk, M.; Deppermann, N.; Borner, K.; Koeppe, P. Quinolone pharmacokinetics and metabolism. *J. Antimicrob. Chemother.*, **1990**, 26(SB), 41-9.<br>Wood, M.J. Tissue penetration and clinical efficacy of enoxacin in respiratory tract infections. *Clin. Pharmacokinet.*, **1989**, *16*(S1), 38-45.<br>Dobbs, B.R.; Gazeley, L.R.; Campbell, A.J.; Edwards, I.R. The effect of age on the pharmacokinetics of enoxacin. *Eur. J. Clin. Pharmacol.*, **1987**, *33*(1),

		101-4.<br>Somogyi, A.A.; Bochner, F.; Misiak, P.M.; Eldon, M.A.; Toothaker, R.D.; Sedman, A.J.Effects of oral cimetidine or ranitidine on the pharmacokinetics of intravenous enoxacin. *J. Clin. Pharmacol.*, **1993**, *33*(1), 53-6.<br>Somogyi, A.A.; Bochner, F. The absorption and disposition of enoxacin in healthy subjects. *J. Clin. Pharmacol.*, **1988**, *28*(8), 707-13.<br>Jones, R.N.; Barry, A.L. *In vitro* evaluation of WIN 57273, a new broad- spectrum fluoroquinolone. *Antimicrob. Agents Chemother.*, **1990**, *34*(2), 306-13.<br>Rolston, K.V.; Ho, D.H.; LeBlanc, B.; Bodey, G.P. *In vitro* evaluation of S-25930 and S-25932, two new quinolones, against aerobic gram-negative isolates from cancer patients. *Antimicrob. Agents Chemother.*, **1987**, *31*(1), 102-3.<br>Gooding, B.B.; Jones, R.N. *In vitro* antimicrobial activity of CP-99,219, a novel azabicyclo-naphthyridone. *Antimicrob. Agents Chemother.*,**1993**, *37*(2), 349-353.
Fleroxacin	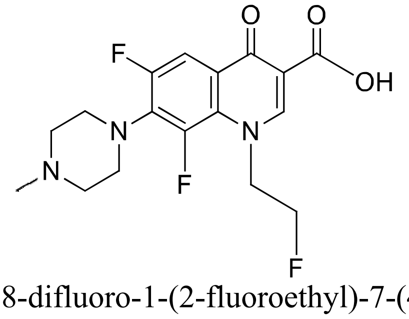<br>6,8-difluoro-1-(2-fluoroethyl)-7-(4-methylpiperazin-1-yl)-4-oxo-1,4-dihydroquinoline-3-carboxylic acid	Nightingale, C.H. Overview of the pharmacokinetics of fleroxacin. *Am. J. Med.*, **1993**, *94*(3A), 38S-43S.<br>Weidekamm, E. Pharmacokinetics of fleroxacin in renal impairment. *Am. J. Med.*, **1993**, *94*(3A), 70S-74S.<br>Sorgel, F.; Metz, R.; Naber, K.; Seelmann, R.; Muth, P. Pharmacokinetics and body fluid penetration of fleroxacin in healthy volunteers. *J. Antimicrob. Chemother.*, **1988**, *22*(SD), 155-67.<br>Reigner, B.G.; Welker, H.A. Factors influencing elimination and distribution of fleroxacin: metaanalysis of individual data from 10 pharmacokinetic studies. *Antimicrob. Agents Chemother.*, **1996**, *40*(3), 575-80.<br>Blouin, R.A.; Hamelin, B.A.; Smith, D.A.; Foster, T.S.; John, W.J.; Welker,

		H.A. Fleroxacin pharmacokinetics in patients with liver cirrhosis. *Antimicrob.*

		Agents Chemother., **1992**, *36*(3), 632-8.<br>Weidekamm, E.; Portmann, R.; Suter, K.; Partos, C.; Dell, D.; Lücker, P.W. Single- and multiple-dose pharmacokinetics of fleroxacin, a trifluorinated quinolone, in humans. *Antimicrob. Agents Chemother.*, **1987**, *31*(12), 1909-14.<br>Gargallo-Viola, D.; Ferrer, S.; Tudela, E.; Robert, M.; Coll, R.; Roser, R, Guinea J. Antibacterial activities and pharmacokinetics of E-4767 and E-5065, two new 8-chlorofluoroquinolones with a 7-azetidin ring substituent. *Antimicrob. Agents Chemother.*, **2001**, *45*(11), 3113-21.<br>Neu, H.C.; Fang, W.; Gu, J.W.; Chin, N.X. *In vitro* activity of OPC-17116. *Antimicrob. Agents Chemother.*, **1992**, *36*(6), 1310-5.<br>Guinea, J.; Robert, M.; Gargallo-Viola, D.; Xicota, M.A.; Garcia, J.; Tudela, E.; Esteve, M.; Coll, R.; Pares, M.; Roser, R. *In vitro* and *in vivo* antibacterial activities of E-4868, a new fluoroquinolone with a 7-azetidin ring substituent. *Antimicrob. Agents Chemother.*, **1993**, *37*(4), 868-74.<br>Jones, R.N.; Barry, A.L. *In vitro* evaluation of WIN 57273, a new broad-spectrum fluoroquinolone. *Antimicrob. Agents Chemother.*, **1990**, *34*(2), 306-13.<br>Bongaerts, G.P.A.; Hoogkamp, J.A.A. *In vitro* activities of BAY Y3118, ciprofloxacin, ofloxacin, and fleroxacin against gram-positive and gram-negative pathogens from respiratory tract and soft tissue infections. *Antimicrob. Agents Chemother.*, **1993**, *37*(9), 2017-9.
Garenoxacin	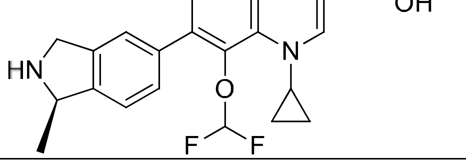	Hayakawa, H.; Fukushima, Y.; Kato, H.; Fukumoto, H.; Kadota, T.; Yamamoto, H.; Kuroiwa,
	(R)-1-cyclopropyl-8-(difluoromethoxy)-7-(1-methylisoindolin-5-yl)-4-oxo-1,4-dihydroquinoline-3-carboxylic acid	H.; Nishigaki, J.; Tsuji, A. Metabolism<br>and disposition of novel des-fluoro

	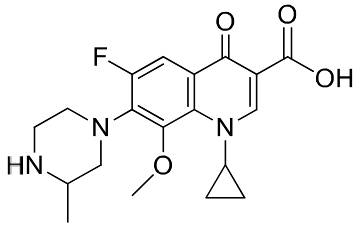	quinolone garenoxacin in experimental animals and interspecies scaling of pharmacokinetic parameters. *Drug Metab. Dispos.*, 2003, *31*(11), 1409-18.<br>Gajjar, D.A.; Bello, A.; Ge, Z.; Christopher, L.; Grasela, D.M. Multiple-dose safety and pharmacokinetics of oral garenoxacin in healthy subjects. *Antimicrob. Agents Chemother.*, **2003**, *47*(7), 2256-63.<br>Tanaka, M.; Yamazaki, E.; Chiba, M.; Yoshihara, K.; Akasaka, T.; Takemura, M.; Sato, K. *In vitro* antibacterial activities of DQ-113, a potent quinolone, against clinical isolates. *Antimicrob. Agents Chemother.*, **2002**, *46*(3), 904-8.<br>Cormican, M.G.; Jones, R.N. Antimicrobial activity and spectrum of LB20304, a novel fluoronaphthyridone. *Antimicrob. Agents Chemother.*, **1997**, *41*(1), 204-11.<br>Hoellman, D.B.; Kelly, L.M.; Jacobs, M.R.; Appelbaum, P.C. Comparative antianaerobic activity of BMS 284756. *Antimicrob. Agents Chemother.*, **2001**, *45*(2), 589-92.<br>Snydman, D.R.; Jacobus, N.V.; McDermott, L.A.; Ruthazer, R.; Goldstein, E.; Finegold, S.; Harrell, L.; Hecht, D.W.; Jenkins, S.; Pierson, C.; Venezia, R.; Rihs, J; Gorbach, S.L. *In vitro* activities of newer quinolones against bacteroides group organisms. *Antimicrob. Agents Chemother.*, **2002**, *46*(10), 3276-9.<br>Otani, T.; Tanaka, M.; Ito, E.; Kurosaka, Y.; Murakami, Y.; Onodera, K.; Akasaka, T.; Sato, K. *In vitro* and *in vivo* antibacterial activities of DK-507k, a

		novel fluoroquinolone. *Antimicrob. Agents Chemother.*, **2003**, *47*(12), 3750-9.
Gatifloxacin		Lober, S.; Ziege, S.; Rau, M.; Schreiber, G.; Mignot, A.; Koeppe, P.; Lode, H.

	1-cyclopropyl-6-fluoro-8-methoxy-7-(3-methylpiperazin-1-yl)-4-oxo-1,4- dihydroquinoline-3-carboxylic acid	Pharmacokinetics of gatifloxacin and interaction with an antacid containing aluminum and magnesium. *Antimicrob. Agents Chemother.*, **1999**, *43*(5), 1067-71.<br>Lubasch, A.; Keller, I.; Borner, K.; Koeppe, P.; Lode, H. Comparative pharmacokinetics of ciprofloxacin, gatifloxacin, grepafloxacin, levofloxacin, trovafloxacin, and moxifloxacin after single oral administration in healthy volunteers. *Antimicrob. Agents Chemother.*, **2000**, *44*(10), 2600-3.<br>Boy, D.; Well, M.; Kinzig-Schippers, M.; Sörgel, F.; Ankel-Fuchs, D.; Naber, K.G. Urinary bactericidal activity, urinary excretion and plasma concentrations of gatifloxacin (400 mg) versus ciprofloxacin (500 mg) in healthy volunteers after a single oral dose. *Int. J. Antimicrob. Agents*, **2004**, *23*(S1), S6-16.<br>Tanaka, M.; Yamazaki, E.; Chiba, M.; Yoshihara, K.; Akasaka, T.; Takemura, M.; Sato, K. *In vitro* antibacterial activities of DQ-113, a potent quinolone, against clinical isolates. *Antimicrob. Agents Chemother.*, **2002**, *46*(3), 904-8.<br>Milatovic, D.; Schmitz, F.J.; Brisse, S.; Verhoef, J.; Fluit, A.C. *In vitro* activities of sitafloxacin (DU-6859a) and six other fluoroquinolones against 8,796 clinical bacterial isolates. *Antimicrob. Agents Chemother.*, **2000**, *44*(4), 1102-7.<br>Almer, L.S.; Hoffrage, J.B.; Keller, E.L.; Flamm, R.K.; Shortridge, V.D. *In vitro* and bactericidal activities of ABT-492, a novel fluoroquinolone, against Gram-positive and Gram-negative organisms. *Antimicrob. Agents Chemother.*, **2004**, *48*(7), 2771-7.<br>Yong, D.E.; Cheong, H.J.; Kim, Y.S.; Park, Y.J.; Kim, W.J.; Woo, J.H.; Lee, K.W.; Kang, M.W.; Choo,

		Y.S. *In vitro* activity of gemifloxacin against recent clinical isolates of bacteria in Korea. *J. Korean Med. Sci.*, **2002**, *17(6)*, 737-42.<br>Fung-Tomc, J.; Minassian, B.; Kolek, B.; Washo, T.; Huczko, E.; Bonner, D. *In vitro* antibacterial spectrum of a new

		broad-spectrum 8-methoxy fluoroquinolone, gatifloxacin. *J. Antimicrob. Chemother.*, **2000**, *45*(4), 437-46.<br>Jones, R.N.; Croco, M.A.; Pfaller, M.A.; Beach, M.L.; Kugler, K.C. Antimicrobial activity evaluations of gatifloxacin, a new fluoroquinolone: contemporary pathogen results from a global antimicrobial resistance surveillance program (SENTRY, 1997). *Clin. Microbiol. Infect.*, **1999**, *5*(9), 540-6.<br>Otani, T.; Tanaka, M.; Ito, E.; Kurosaka, Y.; Murakami, Y.; Onodera, K.; Akasaka, T.; Sato, K. *In vitro* and *in vivo* antibacterial activities of DK-507k, a novel fluoroquinolone. *Antimicrob. Agents Chemother.*, **2003**, *47*(12), 3750-9.<br>Roychoudhury, S.; Twinem, T.L.; Makin, K.M.; McIntosh, E.J.; Ledoussal, B.; Catrenich, C.E. Activity of non-fluorinated quinolones (NFQs) against quinolone-resistant *Escherichia coli* and *Streptococcus pneumoniae*. *J. Antimicrob. Chemother.*, **2001**, *48*(1), 29-36.<br>Fukuda, H.; Hori, S.; Hiramatsu, K. Antibacterial activity of gatifloxacin (AM-1155, CG5501, BMS-206584), a newly developed fluoroquinolone, against sequentially acquired quinolone-resistant mutants and the norA transformant of *Staphylococcus aureus*. *Antimicrob. Agents Chemother.*, **1998**, *42*(8), 1917-22.<br>Perry, C.M.; Barman Balfour, J.A.; Lamb, H.M. Gatifloxacin. *Drugs*, **1999**, *58*(4), 683-96.

Gemifloxacin	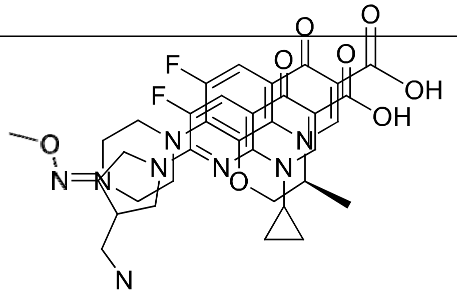 (Z)-7-(3-(aminomethyl)-4- (methoxy-imino)pyrrolidin-1-yl)-1- cyclopropyl-6-fluoro-4-oxo-1,4-dihydro- 1,8-naphthyridine-3-carboxylic acid	Allen, A.; Bygate, E.; Vousden, M.; Oliver, S.; Johnson, M.; Ward, C.; Cheon, A.; Choo, Y.S.; Kim, I. Multiple-dose pharmacokinetics and tolerability of gemifloxacin administered orally to healthy volunteers. *Antimicrob. Agents Chemother.*, **2001**, *45*(2), 540-5. Tanaka, M.; Yamazaki, E.; Chiba, M.; Yoshihara, K.; Akasaka, T.; Takemura, M.; Sato, K. *In vitro* antibacterial activities of DQ-113, a potent quinolone, against clinical isolates. *Antimicrob. Agents Chemother.*, **2002**, *46*(3), 904-8. Yun, H.J.; Min, Y.H.; Lim, J.A.; Kang, J.W.; Kim, S.Y.; Kim, M.J.; Jeong, J.H.; Choi, Y.J.; Kwon, H.J.; Jung, Y.H.; Shim, M.J.; Choi, E.C. *In vitro* and *in vivo* antibacterial activities of DW286, a new fluoronaphthyridone antibiotic. *Antimicrob. Agents Chemother.*, **2002**, *46*(9), 3071-4. Yong, D.E.; Cheong, H.J.; Kim, Y.S.; Park, Y.J.; Kim, W.J.; Woo, J.H.; Lee, K.W.; Kang, M.W.; Choo, Y.S. *In vitro* activity of gemifloxacin against recent clinical isolates of bacteria in Korea. *J. Korean Med. Sci.*, **2002**, *17(6)*, 737-42.
Grepafloxacin	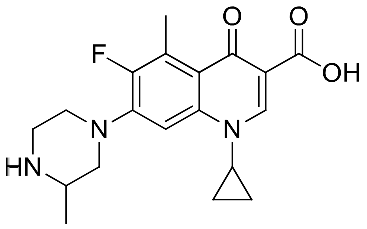 1-cyclopropyl-6-fluoro-5-methyl-7-(3-methylpiperazin-1-yl)-4-oxo-1,4- dihydroquinoline-3-carboxylic acid	Efthymiopoulos, C.; Bramer, S.L.; Maroli, A. Pharmacokinetics of grepafloxacin after oral administration of single and repeat doses in healthy young males. *Clin. Pharmacokinet.*, **1997**, *33*(S1), 1-8. Pea, F.; Pavan, F.; Lugatti, E.; Dolcet, F.; Talmassons, G.; Screm, M.C.; Furlanut, M. Pharmacokinetic and pharmacodynamic aspects of oral moxifloxacin 400 mg/day in elderly patients with acute exacerbation of chronic bronchitis. *Clin. Pharmacokinet.*, **2006**, *45*(3), 287-95. Neu, H.C.; Fang, W.; Gu, J.W.; Chin, N.X. *In vitro* activity of OPC-17116. *Antimicrob. Agents Chemother.*, **1992**, *36*(6), 1310-5.
Levofloxacin		Chien, S.C.; Wong, F.A.; Fowler, C.L.; Callery-D'Amico, S.V.; Williams, R.R.; Nayak, R.; Chow, A.T.

	(S)-9-fluoro-3-methyl-10-(4- methylpiperazin-1-yl)-7-oxo-3,7-dihydro- 2H-[1,4]oxazino[2,3,4-ij]quinoline-6- carboxylic acid	Double-blind evaluation of the safety and pharmacokinetics of multiple oral once-daily 750-milligram and 1-gram doses of levofloxacin in healthy volunteers. *Antimicrob. Agents Chemother.*, **1998**, *42*(4), 885-8.<br>Chien, S.C.; Rogge, M.C.; Gisclon, L.G.; Curtin, C.; Wong, F.; Natarajan, J.; Williams, R.R.; Fowler, C.L.; Cheung, W.K.; Chow, A.T. Pharmacokinetic profile of levofloxacin following once-daily 500-milligram oral or intravenous doses. *Antimicrob. Agents Chemother.*, **1997**, *41*(10), 2256-60.<br>Okazaki, O.; Kojima, C.; Hakusui, H.; Nakashima, M. Enantioselective disposition of ofloxacin in humans. *Antimicrob. Agents Chemother.*, **1991**, *35*(10), 2106-9.<br>Lubasch, A.; Keller, I.; Borner, K.; Koeppe, P.; Lode, H. Comparative pharmacokinetics of ciprofloxacin, gatifloxacin, grepafloxacin, levofloxacin, trovafloxacin, and moxifloxacin after single oral administration in healthy volunteers. *Antimicrob. Agents Chemother.*, **2000**, *44*(10), 2600-3.<br>Geerdes-Fenge, H.F.; Wiedersich, A.; Wagner, S.; Lehr, K.H.; Koeppe, P.; Lode, H. Levofloxacin pharmacokinetics and serum bactericidal activities against five enterobacterial species. *Antimicrob. Agents Chemother.*, **2000**, *44*(12), 3478-80.<br>Tanigawara, Y.; Nomura, H.; Kagimoto, N.; Okumura, K.; Hori, R. Premarketing population pharmacokinetic study of levofloxacin in normal subjects and patients with infectious diseases. *Biol. Pharm. Bull.*, **1995**, *18*(2), 315-20.<br>Fish, D.N.; Chow, A.T. The clinical pharmacokinetics of levofloxacin. *Clin. Pharmacokinet.*, **1997**, *32*(2), 101-19.<br>Azanza, J.R.; Sádaba, B.; Quetglas,
		E.G.; Escolar, M. Levofloxacino. *Rev. Med. Univ. Navarra*, **1998**, *42*(4), 220-5.<br>Davis, R.; Bryson, H.M. Levofloxacin. A review of its antibacterial activity,

		pharmacokinetics and therapeutic efficacy. *Drugs*, **1994**, *47*(4), 677-700.<br>Cormican, M.G.; Jones, R.N. Antimicrobial activity and spectrum of LB20304, a novel fluoronaphthyridone. *Antimicrob. Agents Chemother.*, **1997**, *41*(1), 204-11.<br>Goldstein, E.J.; Citron, D.M.; Hudspeth, M.; Hunt Gerardo, S.; Merriam, C.V. Trovafloxacin compared with levofloxacin, ofloxacin, ciprofloxacin, azithromycin and clarithromycin against unusual aerobic and anaerobic human and animal bite-wound pathogens. *J. Antimicrob. Chemother.*, **1998**, *41*(3), 391-6.<br>Tanaka, M.; Yamazaki, E.; Chiba, M.; Yoshihara, K.; Akasaka, T.; Takemura, M.; Sato, K. *In vitro* antibacterial activities of DQ-113, a potent quinolone, against clinical isolates. *Antimicrob. Agents Chemother.*, **2002**, *46*(3), 904-8.<br>Brueggemann, A.B.; Kugler, K.C.; Doern, G.V. *In vitro* activity of BAY 12-8039, a novel 8-methoxyquinolone, compared to activities of six fluoroquinolones against *Streptococcus pneumoniae*, *Haemophilus influenzae*, and *Moraxella catarrhalis*. *Antimicrob. Agents Chemother.*, **1997**, *41*(7), 1594-7.<br>Montanari, M.P.; Mingoia, M.; Varaldo, P.E. *In vitro* antibacterial activities of AF 3013, the active metabolite of prulifloxacin, against nosocomial and community Italian isolates. *Antimicrob. Agents Chemother.*, **2001**, *45*(12), 3616-22.<br>Hoellman, D.B.; Kelly, L.M.; Jacobs, M.R.; Appelbaum, P.C. Comparative

		antianaerobic activity of BMS 284756. *Antimicrob. Agents Chemother.*, **2001**, *45*(2), 589-92.<br>Milatovic, D.; Schmitz, F.J.; Brisse, S.; Verhoef, J.; Fluit, A.C. *In vitro* activities of sitafloxacin (DU-6859a) and six other fluoroquinolones against 8,796 clinical

	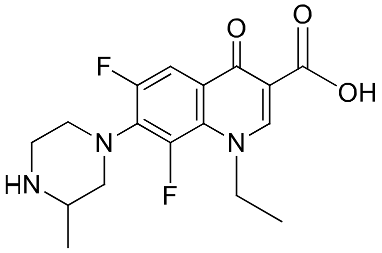	bacterial isolates. *Antimicrob. Agents Chemother.*, **2000**, *44*(4), 1102-7.<br>Coll, R.; Gargallo-Viola, D.; Tudela, E.; Xicota, M.A.; Llovera, S.; Guinea, J. Antibacterial activity and pharmacokinetics of four new 7-azetidinyl fluoroquinolones. *Antimicrob. Agents Chemother.*, **1996**, *40*(1), 274-7.<br>Almer, L.S.; Hoffrage, J.B.; Keller, E.L.; Flamm, R.K.; Shortridge, V.D. *In vitro* and bactericidal activities of ABT-492, a novel fluoroquinolone, against Gram-positive and Gram-negative organisms. *Antimicrob. Agents Chemother.*, **2004**, *48*(7), 2771-7.<br>Nilius, A.M.; Shen, L.L.; Hensey-Rudloff, D.; Almer, L.S.; Beyer, J.M.; Balli, D.J.; Cai, Y.; Flamm, R.K. *In vitro* antibacterial potency and spectrum of ABT-492, a new fluoroquinolone. *Antimicrob. Agents Chemother.*, **2003**, *47*(10), 3260-9.<br>Yong, D.E.; Cheong, H.J.; Kim, Y.S.; Park, Y.J.; Kim, W.J.; Woo, J.H.; Lee, K.W.; Kang, M.W.; Choo, Y.S. *In vitro* activity of gemifloxacin against recent clinical isolates of bacteria in Korea. *J. Korean Med. Sci.*, **2002**, *17*(6), 737-42.<br>Barry, A.L.; Fuchs, P.C.; Brown, S.D. *In vitro* activities of three nonfluorinated quinolones against representative bacterial isolates. *Antimicrob. Agents Chemother.*, **2001**, *45*(6), 1923-7.<br>Rolston, K.V.; Ho, D.H.; LeBlanc, B.; Bodey, G.P. *In vitro* evaluation of S-25930 and S-25932, two new quinolones, against aerobic gram-negative isolates from cancer patients. *Antimicrob. Agents Chemother.*, **1987**, *31*(1), 102-3.<br>Otani, T.; Tanaka, M.; Ito, E.; Kurosaka,
		Y.; Murakami, Y.; Onodera, K.; Akasaka, T.; Sato, K. *In vitro* and *in vivo* antibacterial activities of DK-507k, a novel fluoroquinolone. *Antimicrob. Agents Chemother.*, **2003**, *47*(12), 3750-9.
Lomefloxacin		Freeman, C.D.; Nicolau, D.P.; Belliveau, P.P.; Nightingale, C.H. Lomefloxacin clinical pharmacokinetics. *Clin.*

	1-ethyl-6,8-difluoro-7-(3-methylpiperazin-1-yl)-4-oxo-1,4-dihydroquinoline-3- carboxylic acid 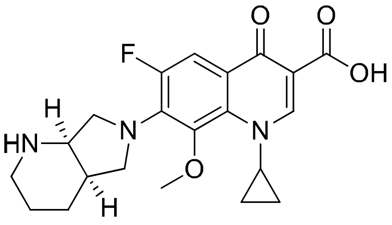	*Pharmacokinet.*, **1993**, *25*(1), 6-19. Wadworth, A.N.; Goa, K.L. Lomefloxacin. A review of its antibacterial activity, pharmacokinetic properties and therapeut ic use. *Drugs*, **1991**, *42*(6), 1018-60. Nightingale, C.H. Overview of the pharmacokinetics of fleroxacin. *Am. J. Med.*, **1993**, *94*(3A), 38S-43S. Neu, H.C.; Fang, W.; Gu, J.W.; Chin, N.X. *In vitro* activity of OPC-17116. *Antimicrob. Agents Chemother.*, **1992**, *36*(6), 1310-5. Kim, J.H.; Choi, K.H.; Kim, J.W.; Lee, J.H.; Choi, E.C.; Kim, B.K. *In-vitro* and *in-vivo* antibacterial activity of CFC-222, a new fluoroquinolone. *J. Antimicrob. Chemother.*, **1998**, *41*(2), 223-9. Clement, J.J.; Tanaka, S.K.; Alder, J.; Vojtko, C.; Beyer, J.; Hensey, D.; Ramer, N.; McDaniel, D.; Chu, D.T. *In vitro* and *in vivo* evaluations of A-80556, a new fluoroquinolone. *Antimicrob. Agents Chemother.*, **1994**, *38*(5), 1071-8. Bongaerts, G.P.A.; Hoogkamp, J.A.A. *In vitro* activities of BAY Y3118, ciprofloxacin, ofloxacin, and fleroxacin against gram-positive and gram-negative pathogens from respiratory tract and soft tissue infections. *Antimicrob. Agents Chemother.*, **1993**, *37*(9), 2017-9. Venezia, R.A.; Prymas, L.A.; Shayegani, A.; Yocum, D.M. *In vitro* activities of amifloxacin and two of its metabolites. *Antimicrob. Agents Chemother.*, **1989**, *33*(5), 762-6.
Merafloxacin	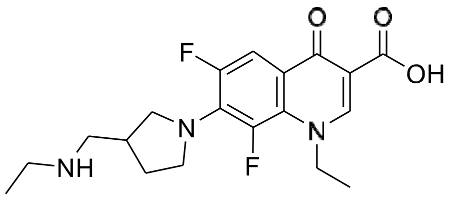 1-ethyl-7-(3- ((ethylamino)methyl)pyrrolidin-1-yl)-6,8- difluoro-4-oxo-1,4-dihydroquinoline-3-carboxylic acid	Chapman, J.S.; Georgopapadakou, N.H. Routes of quinolone permeation in *Escherichia coli. Antimicrob. Agents Chemother.*, **1988**, *32*(4), 438-42.
Moxifloxacin		Stass, H.; Dalhoff, A.; Kubitza, D.; Schühly, U. Pharmacokinetics, safety, and tolerability of ascending single doses of moxifloxacin, a new 8-methoxy quinolone, administered to healthy

	1-cyclopropyl-6-fluoro-7-((4aR,7aR)-hexahydro-1H-pyrrolo[3,4-b]pyridin-6(2H)-yl)-8-methoxy-4-oxo-1,4-dihydroquinoline-3-carboxylic acid	subjects. *Antimicrob. Agents Chemother.*, **1998**, *42*(8), 2060-5.<br>Siefert, H.M.; Domdey-Bette, A.; Henninger, K.; Hucke, F.; Kohlsdorfer, A.; Stass, H.H. Pharmacokinetics of the 8-methoxyquinolone, moxifloxacin: a comparison in humans and other mammalian species. *J. Antimicrob. Chemother.*, **1999**, *43*(SB), 69-76.<br>Stass, H.; Kubitza, D. Pharmacokinetics and elimination of moxifloxacin after oral and intravenous administration in man. *J. Antimicrob. Chemother.*, **1999**, *43*(SB), 83-90.<br>Stass, H.; Kubitza, D.; Halabi, A.; Delesen, H. Pharmacokinetics of moxifloxacin, a novel 8-methoxy-quinolone, in patients with renal dysfunction. *Br. J. Clin. Pharmacol.*, **2002**, *53*(3), 232-7.<br>Burkhardt, O.; Borner, K.; Stass, H.; Beyer, G.; Allewelt, M.; Nord, C.E.; Lode, H. Single- and multiple-dose pharmacokinetics of oral moxifloxacin and clarithromycin, and concentrations in serum, saliva and faeces. *Scand. J. Infect. Dis.*, **2002**, *34*(12), 898-903.<br>Stass, H.; Schühly, U.; Möller, J.G.; Delesen, H. Effects of sucralfate on the oral bioavailability of moxifloxacin, a novel 8-methoxyfluoroquinolone, in healthy volunteers. *Clin. Pharmacokinet.*, **2001**, *40*(S1), 49-55.<br>Stass, H.; Kubitza, D. Effects of iron

		supplements on the oral bioavailability of moxifloxacin, a novel 8-methoxyfluoroquinolone, in humans. *Clin. Pharmacokinet.*, **2001**, *40*(S1), 57-62.<br>Stass, H.; Kubitza, D. Effects of dairy products on the oral bioavailability of moxifloxacin, a novel 8-methoxyfluoroquinolone, in healthy volunteers. *Clin. Pharmacokinet.*, **2001**, *40*(S1), 33-8.<br>Stass, H.; Wandel, C.; Delesen, H.; Möller, J.G. Effect of calcium supplements on the oral bioavailability of moxifloxacin in healthy male volunteers. *Clin. Pharmacokinet.*, **2001**, *40*(S1), 27-

		32.<br>Lubasch, A.; Keller, I.; Borner, K.; Koeppe, P.; Lode, H. Comparative pharmacokinetics of ciprofloxacin, gatifloxacin, grepafloxacin, levofloxacin, trovafloxacin, and moxifloxacin after single oral administration in healthy volunteers. *Antimicrob. Agents Chemother.*, **2000**, *44*(10), 2600-3.<br>Tanaka, M.; Yamazaki, E.; Chiba, M.; Yoshihara, K.; Akasaka, T.; Takemura, M.; Sato, K. *In vitro* antibacterial activities of DQ-113, a potent quinolone, against clinical isolates. *Antimicrob. Agents Chemother.*, **2002**, *46*(3), 904-8.<br>Montanari, M.P.; Mingoia, M.; Varaldo, P.E. *In vitro* antibacterial activities of AF 3013, the active metabolite of prulifloxacin, against nosocomial and community Italian isolates. *Antimicrob. Agents Chemother.*, **2001**, *45*(12), 3616-22.<br>Brueggemann, A.B.; Kugler, K.C.; Doern, G.V. *In vitro* activity of BAY 12-8039, a novel 8-methoxyquinolone, compared to activities of six fluoroquinolones against *Streptococcus pneumoniae*, *Haemophilus influenzae*, and *Moraxella catarrhalis*. *Antimicrob. Agents Chemother.*, **1997**, *41*(7), 1594-7.

	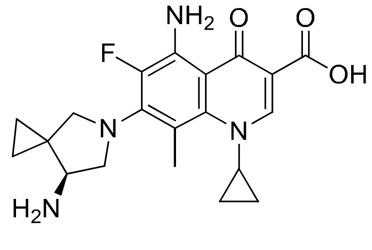	Hoellman, D.B.; Kelly, L.M.; Jacobs, M.R.; Appelbaum, P.C. Comparative antianaerobic activity of BMS 284756. *Antimicrob. Agents Chemother.*, **2001**, *45*(2), 589-92.<br>Snydman, D.R.; Jacobus, N.V.; McDermott, L.A.; Ruthazer, R.; Goldstein, E.; Finegold, S.; Harrell, L.; Hecht, D.W.; Jenkins, S.; Pierson, C.; Venezia, R.; Rihs, J; Gorbach, S.L. *In vitro* activities of newer quinolones against bacteroides group organisms. *Antimicrob. Agents Chemother.*, **2002**, *46*(10), 3276-9.<br>Milatovic, D.; Schmitz, F.J.; Brisse, S.; Verhoef, J.; Fluit, A.C. *In vitro* activities of sitafloxacin (DU-6859a) and six other fluoroquinolones against 8,796 clinical bacterial isolates. *Antimicrob. Agents Chemother.*, **2000**, *44*(4), 1102-7.<br>Almer, L.S.; Hoffrage, J.B.; Keller, E.L.; Flamm, R.K.; Shortridge, V.D. *In vitro* and bactericidal activities of ABT- 492, a novel fluoroquinolone, against Gram-positive and Gram-negative organisms. *Antimicrob. Agents Chemother.*, **2004**, *48*(7), 2771-7.<br>Yong, D.E.; Cheong, H.J.; Kim, Y.S.; Park, Y.J.; Kim, W.J.; Woo, J.H.; Lee, K.W.; Kang, M.W.; Choo, Y.S. *In vitro* activity of gemifloxacin against recent clinical isolates of bacteria in Korea. *J. Korean Med. Sci.*, **2002**, *17*(6), 737-42.<br>Otani, T.; Tanaka, M.; Ito, E.; Kurosaka, Y.; Murakami, Y.; Onodera, K.; Akasaka, T.; Sato, K. *In vitro* and *in vivo* antibacterial activities of DK-507k, a novel fluoroquinolone. *Antimicrob. Agents Chemother.*, **2003**, *47*(12), 3750-9.
Norfloxacin	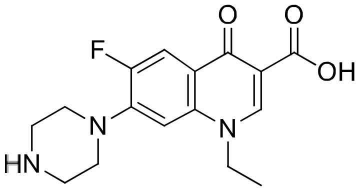<br>1-ethyl-6-fluoro-4-oxo-7-(piperazin-1-yl)-1,4-dihydroquinoline-3-carboxylic acid	Nightingale, C.H. Overview of the pharmacokinetics of fleroxacin. *Am. J. Med.*, **1993**, *94*(3A), 38S-43S.<br>Coll, R.; Gargallo-Viola, D.; Tudela, E.; Xicota, M.A.; Llovera, S.; Guinea, J. Antibacterial activity and pharmacokinetics of four new 7-azetidinyl fluoroquinolones.

		Antimicrob. Agents Chemother., **1996**, *40*(1), 274-7.

	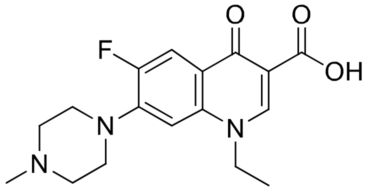	Cunha, B.A.; Qadri, S.M.; Ueno, Y.; Walters, E.A.; Domenico, P. Antibacterial activity of trovafloxacin against nosocomial Gram-positive and Gram-negative isolates. *J. Antimicrob. Chemother.*, **1997**, *39*(SB), 29-34.<br>Jones, R.N.; Barry, A.L. *In vitro* evaluation of WIN 57273, a new broad-spectrum fluoroquinolone. *Antimicrob. Agents Chemother.*, **1990**, *34*(2), 306-13.<br>García-Rodríguez, J.A.; García Sánchez, J.E.; Muñoz Bellido, J.L.; Trujillano, I. *In vitro* activities of irloxacin and E-3846, two new quinolones. *Antimicrob. Agents Chemother.*, **1990**, *34*(6), 1262-7.<br>Gooding, B.B.; Jones, R.N. *In vitro* antimicrobial activity of CP-99,219, a novel azabicyclo-naphthyridone. *Antimicrob. Agents Chemother.*, **1993**, *37*(2), 349-53.<br>Golan, Y.; McDermott, L.A.; Jacobus, N.V.; Goldstein, E.J.; Finegold, S.; Harrell, L.J.; Hecht, D.W.; Jenkins, S.G.; Pierson, C.; Venezia, R.; Rihs, J.; Iannini, P.; Gorbach, S.L.; Snydman, D.R. Emergence of fluoroquinolone resistance among *Bacteroides* species. *J. Antimicrob. Chemother.*, **2003**, *52*(2), 208-13.
Olamufloxacin	(S)-5-amino-7-(7-amino-5- azaspiro[2.4]heptan-5-yl)-1-cyclopropyl-6- fluoro-8-methyl-4-oxo-1,4- dihydroquinoline-3-carboxylic acid	Sun, J.; Deguchi, Y.; Chen, J.M.; Zhang, R.H.; Morimoto, K. Interactions between quinolone antibiotics and phospholipid membrane for prediction of alveolar macrophage uptake *in vitro*. *Acta Pharmacol. Sin.*, **2002**, *23*(5), 430-8.<br>Takahashi, Y.; Masuda, N.; Otsuki, M.; Miki, M.; Nishino, T. *In vitro* activity of HSR-903, a new quinolone. *Antimicrob. Agents Chemother.*, **1997**,*41*(6), 1326-30.

Pazufloxacin	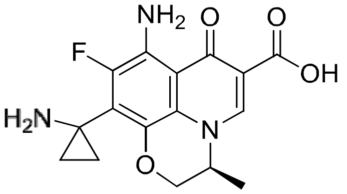<br>(S)-8-amino-10-(1-aminocyclopropyl)-9-fluoro-3-methyl-7-oxo-3,7-dihydro-2H-[1,4]oxazino[2,3,4-ij]quinoline-6-carboxylic acid	Saito, A. Pharmacokinetics of T-3761 in healthy volunteers. *Drugs*, **1995**, *49*(S2), 335-6.<br>Fukuoka, Y.; Ikeda, Y.; Yamashiro, Y.; Takahata, M.; Todo, Y.; Narita, H. *In vitro* and *in vivo* antibacterial activities of T-3761, a new quinolone derivative. *Antimicrob. Agents Chemother.*, **1993**, *37*(3), 384-92.
Pefloxacin	1-ethyl-6-fluoro-7-(4-methylpiperazin-1-yl)-4-oxo-1,4-dihydroquinoline-3- carboxylic acid	Petitjean, O.; Pangon, B.; Brion, N.; Tod, M.; Chaplain, C.; Le Gros, V.; Louchahi, K.; Allouch, P. Pharmacokinetics and bactericidal activities of one 800-milligram dose versus two 400-milligram doses of intravenously administered pefloxacin in healthy volunteers. *Antimicrob. Agents Chemoth.*, **1993**, *37*(4), 737-40.<br>Barre, J.; Houin, G.; Tillement, J.P. Dose-dependent pharmacokinetic study of pefloxacin, a new antibacterial agent, in humans. *J. Pharm. Sci.*, **1984**, *73*(10), 1379-82.<br>Höffler, D.; Schäfer, I.; Koeppe, P.; Sörgel, F. Pharmacokinetics of pefloxacin in normal and impaired renal function. *Arzneimittelforschung*, **1988**, *38*(5), 739-43.<br>Cardey, J.; Silvain, C.; Bouquet, S.; Breux, J.P.; Becq-Giraudon, B.; Fourtillan, J.P.; Beauchant, M. Oral pharmacokinetics and ascitic fluid penetration of pefloxacin in cirrhosis. *Eur. J. Clin. Pharmacol.*, **1987**, *33*(5), 469-72.<br>Neuman, M. Clinical pharmacokinetics of the newer antibacterial 4-quinolones. *Clin. Pharmacokinet.*, **1988**, *14*(2), 96-121.<br>García-Rodríguez, J.A.; García Sánchez, J.E.; Muñoz Bellido, J.L.; Trujillano, I. *In vitro* activities of irloxacin and E-3846, two new quinolones. *Antimicrob. Agents Chemother.*, **1990**, *34*(6), 1262-7.

PGE-4175997	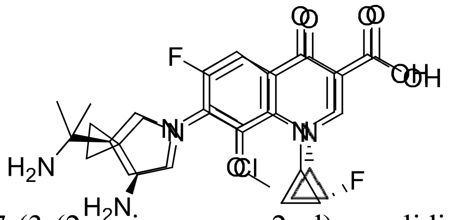<br>(R)-7-(3-(2-aminopropan-2-yl)pyrrolidin-1-yl)-1-cyclopropyl-8-methoxy-4-oxo-1,4-dihydroquinoline-3-carboxylic acid	Barry, A.L.; Fuchs, P.C.; Brown, S.D. *In vitro* activities of three nonfluorinated quinolones against representative bacterial isolates. *Antimicrob. Agents Chemother.*, **2001**, *45*(6), 1923-7.<br>Roychoudhury, S.; Twinem, T.L.; Makin, K.M.; McIntosh, E.J.; Ledoussal, B.; Catrenich, C.E.<br>Activity of non-fluorinated quinolones (NFQs) against quinolone-resistant *Escherichia coli* and *Streptococcus pneumoniae*.<br>*J. Antimicrob. Chemother.*, **2001**, *48*(1), 29-36.
PGE-9262932	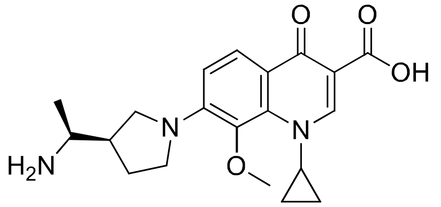<br>7-((R)-3-((S)-1-aminoethyl)pyrrolidin-1-yl)-1-cyclopropyl-8-methoxy-4-oxo-1,4-dihydroquinoline-3-carboxylic acid	Same as for PGE-4175997
PGE-9509924	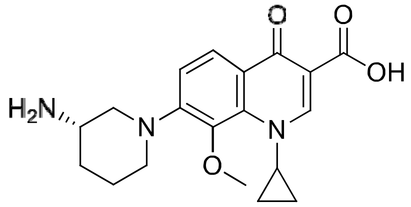<br>(S)-7-(3-aminopiperidin-1-yl)-1-cyclopropyl-8-methoxy-4-oxo-1,4-dihydroquinoline-3-carboxylic acid	Same as for PGE-4175997
Rufloxacin	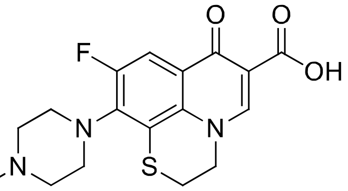<br>9-fluoro-10-(4-methylpiperazin-1-yl)-7-oxo-3,7-dihydro-2H-[1,4]thiazino[2,3,4-ij]quinoline-6-carboxylic acid	Imbimbo, B.P.; Broccali, G.; Cesana, M.; Crema, F.; Attardo-Parrinello, G. Inter- and intrasubject variabilities in the pharmacokinetics of rufloxacin after single oral administration to healthy volunteers. *Antimicrob. Agents Chemother.*, **1991**, *35*(2), 390-3.<br>Perry, G.; Mant, T.G.; Morrison, P.J.; Sacks, S.; Woodcook, J.; Wise, R.; Imbimbo, B.P. Pharmacokinetics of rufloxacin in patients with impaired renal function. *Antimicrob. Agents Chemother.*, **1993**, *37*(4), 637-41.<br>Choi, K.H.; Hong, J.S.; Kim, S.K.; Lee, D.K.; Yoon, S.J.; Choi, E.C. *In-vitro* and *in-vivo* activities of DW-116, a new fluoroquinolone.<br>*J. Antimicrob. Chemother.*, **1997**, *39*(4),
		509-14.
Sitafloxacin		Nakashima, M.; Uematsu, T.; Kosuge,

	7-((S)-7-amino-5-azaspiro[2.4]heptan-5-yl)-8-chloro-6-fluoro-1-((1S,2R)-2-fluorocyclopropyl)-4-oxo-1,4- dihydroquinoline-3-carboxylic acid	K.; Umemura, K.; Hakusui, H.; Tanaka, M. Pharmacokinetics and tolerance of DU-6859a, a new fluoroquinolone, after single and multiple oral doses in healthy volunteers. *Antimicrob. Agents Chemother.*, **1995**, *39*(1), 170-4.<br>O'Grady, J.; Briggs, A.; Atarashi, S.; Kobayashi, H.; Smith, R.L.; Ward, J.; Ward, C.; Milatovic, D. Pharmacokinetics and absolute bioavailability of sitafloxacin, a new fluoroquinolone antibiotic, in healthy male and female Caucasian subjects. *Xenobiotica*, **2001**, *31*(11), 811-22.<br>Tanaka, M.; Yamazaki, E.; Chiba, M.; Yoshihara, K.; Akasaka, T.; Takemura, M.; Sato, K. *In vitro* antibacterial activities of DQ-113, a potent quinolone, against clinical isolates. *Antimicrob. Agents Chemother.*, **2002**, *46*(3), 904-8.<br>Korten, V.; Tomayko, J.F.; Murray, B.E. Comparative *in vitro* activity of DU-6859a, a new fluoroquinolone agent, against gram-positive cocci. *Antimicrob. Agents Chemother.*, **1994**, *38*(3), 611-5.<br>Snydman, D.R.; Jacobus, N.V.; McDermott, L.A.; Ruthazer, R.; Goldstein, E.; Finegold, S.; Harrell, L.; Hecht, D.W.; Jenkins, S.; Pierson, C.; Venezia, R.; Rihs, J; Gorbach, S.L. *In vitro* activities of newer quinolones against bacteroides group organisms. *Antimicrob. Agents Chemother.*, **2002**, *46*(10), 3276-9.<br>Milatovic, D.; Schmitz, F.J.; Brisse, S.; Verhoef, J.; Fluit, A.C. *In vitro* activities of sitafloxacin (DU-6859a) and six other fluoroquinolones against 8,796 clinical bacterial isolates. *Antimicrob. Agents Chemother.*, **2000**, *44*(4), 1102-7.<br>Otani, T.; Tanaka, M.; Ito, E.; Kurosaka, Y.; Murakami, Y.; Onodera, K.; Akasaka, T.; Sato, K. *In vitro* and *in vivo* antibacterial activities of DK-507k, a novel fluoroquinolone. *Antimicrob. Agents Chemother.*, **2003**, *47*(12), 3750-

		9.

Sparfloxacin	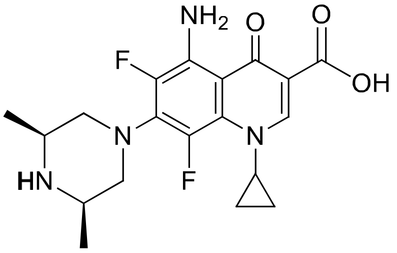<br>5-amino-1-cyclopropyl-7-((3S,5R)-3,5- dimethylpiperazin-1-yl)-6,8-difluoro-4-oxo-1,4-dihydroquinoline-3-carboxylic acid	Trautmann, M.; Ruhnke, M.; Borner, K.; Wagner, J.; Koeppe, P. Pharmacokinetics of sparfloxacin and serum bactericidal activity against pneumococci. *Antimicrob. Agents Chemother.*, **1996**, *40*(3), 776-9.<br>Zix, J.A.; Geerdes-Fenge, H.F.; Rau, M.; Vöckler, J.; Borner, K.; Koeppe, P.; Lode, H. Pharmacokinetics of sparfloxacin and interaction with cisapride and sucralfate. *Antimicrob. Agents Chemother.*, **1997**, *41*(8), 1668-72.<br>Shimada, J.; Nogita, T.; Ishibashi, Y.Clinical pharmacokinetics of sparfloxacin.*Clin. Pharmacokinet.*, **1993**, *25*(5), 358-69.<br>Ritz, M.; Lode, H.; Fassbender, M.; Borner, K.; Koeppe, P.; Nord, C.E. Multiple-dose pharmacokinetics of sparfloxacin and its influence on fecal flora. *Antimicrob. Agents Chemother.*, **1994**, *38*(3), 455-9.<br>Goa, K.L.; Bryson, H.M.; Markham, A. Sparfloxacin. A review of its antibacterial activity, pharmacokinetic properties, clinical efficacy and tolerability in lower respiratory tract infections. *Drugs*, **1997**, *53*(4), 700-25.<br>Gries, J.M.; Honorato, J.; Taburet, A.M.; Alvarez, M.P.; Sadaba, B.; Azanza, J.R.; Singlas, E. Cimetidine does not alter sparfloxacin pharmacokinetics. *Int. J. Clin. Pharmacol. Ther.*, **1995**, *33*(11), 585-7.<br>Montay, G.; Bruno, R.; Vergniol, J.C.; Ebmeier, M.; Le Roux, Y.; Guimart, C.; Frydman, A.; Chassard, D.; Thebault, J.J. Pharmacokinetics of sparfloxacin in humans after single oral administration at doses of 200, 400, 600, and 800 mg. *J. Clin. Pharmacol.*, **1994**, *34*(11), 1071-6.<br>Cormican, M.G.; Jones, R.N. Antimicrobial activity and spectrum

		of LB20304, a novel fluoronaphthyridone. *Antimicrob. Agents Chemother.*, **1997**,

		41(1), 204-11.<br>Goldstein, E.J.; Citron, D.M.; Hunt Gerardo, S.; Hudspeth, M.; Merriam, C.V.<br>Comparative *in vitro* activities of DU-6859a, levofloxacin, ofloxacin, sparfloxacin, and ciprofloxacin against 387 aerobic and anaerobic bite wound isolates. *Antimicrob. Agents Chemother.*, **1997**, *41*(5), 1193-5.<br>Tanaka, M.; Yamazaki, E.; Chiba, M.; Yoshihara, K.; Akasaka, T.; Takemura, M.; Sato, K. *In vitro* antibacterial activities of DQ-113, a potent quinolone, against clinical isolates. *Antimicrob. Agents Chemother.*, **2002**, *46*(3), 904-8.<br>Goldstein, E.J.; Citron, D.M.; Hudspeth, M; Hunt Gerardo, S.; Merriam, C.V.<br>*In vitro* activity of Bay 12-8039, a new 8-methoxyquinolone, compared to the activities of 11 other oral antimicrobial agents against 390 aerobic and anaerobic bacteria isolated from human and animal bite wound skin and soft tissue infections in humans. *Antimicrob. Agents Chemother.*, **1997**, *41*(7), 1552-7.<br>Brueggemann, A.B.; Kugler, K.C.; Doern, G.V.<br>*In vitro* activity of BAY 12-8039, a novel 8-methoxyquinolone, compared to activities of six fluoroquinolones against *Streptococcus pneumoniae*, *Haemophilus influenzae*, and *Moraxella catarrhalis*. *Antimicrob. Agents Chemother.*, **1997**, *41*(7), 1594-7.<br>Yun, H.J.; Min, Y.H.; Lim, J.A.; Kang, J.W.; Kim, S.Y.; Kim, M.J.; Jeong, J.H.; Choi, Y.J.; Kwon, H.J.; Jung, Y.H.; Shim, M.J.; Choi, E.C. *In vitro* and *in vivo* antibacterial activities of DW286, a new fluoronaphthyridone antibiotic.

		Antimicrob. Agents Chemother., **2002**, *46*(9), 3071-4.<br>Montanari, M.P.; Mingoia, M.; Varaldo, P.E. *In vitro* antibacterial activities of AF 3013, the active metabolite of

		prulifloxacin, against nosocomial and community Italian isolates. *Antimicrob. Agents Chemother.*, **2001**, *45*(12), 3616-22.<br>Neu, H.C.; Chin, N.X. *In vitro* activity of the new fluoroquinolone CP-99,219. *Antimicrob. Agents Chemother.*, **1994**, *38*(11), 2615-22.<br>Jones, R.N.; Croco, M.A.; Pfaller, M.A.; Beach, M.L.; Kugler, K.C. Antimicrobial activity evaluations of gatifloxacin, a new fluoroquinolone: contemporary pathogen results from a global antimicrobial resistance surveillance program (SENTRY, 1997). *Clin. Microbiol. Infect.*, **1999**, *5*(9), 540-6.<br>Kim, J.H.; Choi, K.H.; Kim, J.W.; Lee, J.H.; Choi, E.C.; Kim, B.K. *In-vitro* and *in-vivo* antibacterial activity of CFC-222, a new fluoroquinolone. *J. Antimicrob. Chemother.*, **1998**, *41*(2), 223-9.<br>Choi, K.H.; Hong, J.S.; Kim, S.K.; Lee, D.K.; Yoon, S.J.; Choi, E.C. *In-vitro* and *in-vivo* activities of DW-116, a new fluoroquinolone. *J. Antimicrob. Chemother.*, **1997**, *39*(4), 509-14.<br>Clement, J.J.; Tanaka, S.K.; Alder, J.; Vojtko, C.; Beyer, J.; Hensey, D.; Ramer, N.; McDaniel, D.; Chu, D.T. *In vitro* and *in vivo* evaluations of A-80556, a new fluoroquinolone. *Antimicrob. Agents Chemother.*, **1994**, *38*(5), 1071-8.<br>Chin, N.X.; Gu, J.W.; Yu, K.W.; Zhang, Y.X.; Neu, H.C. *In vitro* activity of sparfloxacin. *Antimicrob. Agents Chemother.*, **1991**, *35*(3), 567-71.<br>Borobio, M.V.; Conejo, M.; Ramirez, E.; Suarez, A.I.; Perea, E.J. Comparative activities of eight quinolones against members of the Bacteroides fragilis

	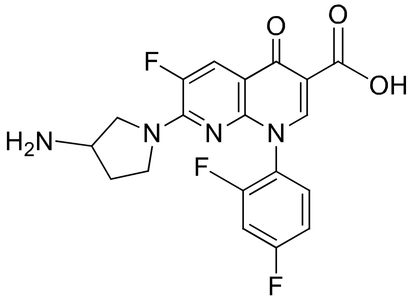	group. *Antimicrob. Agents Chemother.*, **1994**, *38*(6), 1442-5.<br>Wexler, H.M.; Molitoris, E.; Finegold, S.M. *In vitro* activity of BayY3118 against anaerobic bacteria. *Antimicrob. Agents Chemother.*, **1993**, *37*(11), 2509-13.<br>Goldstein, E.J.; Citron, D.M. Comparative activity of ciprofloxacin, ofloxacin, sparfloxacin, temafloxacin, CI-960, CI-990, and WIN 57273 against anaerobic bacteria. *Antimicrob. Agents Chemother.*, **1992**, *36*(5), 1158-62.
Temafloxacin	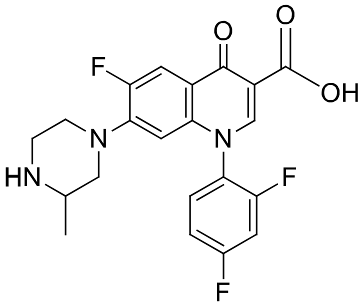<br>1-(2,4-difluorophenyl)-6-fluoro-7-(3-methylpiperazin-1-yl)-4-oxo-1,4- dihydroquinoline-3-carboxylic acid	Granneman, G.R.; Braeckman, R.; Kraut, J.; Shupien, S.; Craft, J.C. Temafloxacin pharmacokinetics in subjects with normal and impaired renal function. *Antimicrob. Agents Chemother.*, **1991**, *35*(11), 2345-51.<br>Granneman, G.R.; Carpentier, P.; Morrison, P.J.; Pernet, A.G. Pharmacokinetics of temafloxacin in humans after single oral doses. *Antimicrob. Agents Chemother.*, **1991**, *35*(3), 436-41.<br>Andersson, M.I.; MacGowan, A.P. Development of the quinolones. *J Antimicrob Chemother.*, **2003**, *51*(S1), 1-11.<br>Piddock, L.J.; Jin, Y.F.; Griggs, D.J. Effect of hydrophobicity and molecular mass on the accumulation of fluoroquinolones by *Staphylococcus aureus*. *J. Antimicrob. Chemother.*, **2001**, *47*(3), 261-70.<br>Neu, H.C.; Chin, N.X. *In vitro* activity of the new fluoroquinolone CP-99,219. *Antimicrob. Agents Chemother.*, **1994**, *38*(11), 2615-22.<br>Neu, H.C.; Fang, W.; Gu, J.W.; Chin, N.X. *In vitro* activity of OPC-17116. *Antimicrob. Agents Chemother.*, **1992**, *36*(6), 1310-5.<br>Goldstein, E.J.; Citron, D.M. Comparative activity of ciprofloxacin, ofloxacin, sparfloxacin, temafloxacin, CI-960, CI-990, and WIN 57273 against anaerobic bacteria. *Antimicrob. Agents Chemother.*, **1992**, *36*(5), 1158-62.
Tosufloxacin		Minami, R.; Nakamura, C.; Inotsume,

	7-(3-aminopyrrolidin-1-yl)-1-(2,4-difluorophenyl)-6-fluoro-4-oxo-1,4-dihydro-1,8-naphthyridine-3-carboxylic acid<br>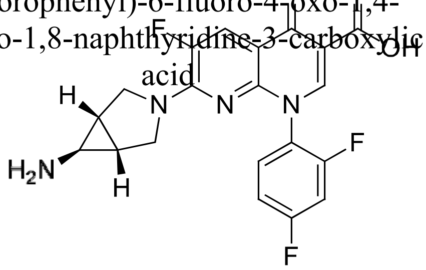 	N.; Nakano, M. Effects of aluminum hydroxide and famotidine on bioavailability of tosufloxacin in healthy volunteers. *Antimicrob. Agents Chemother.*, **1998**, *42*(2), 453-5.<br>Minami, R.; Inotsume, N.; Nakamura, C.; Nakano, M. Stereoselective analysis of the disposition of tosufloxacin enantiomers in man. *Eur. J. Clin. Pharmacol.*, **1993**, *45*(5), 489-91.<br>Piddock, L.J.; Jin, Y.F.; Griggs, D.J. Effect of hydrophobicity and molecular mass on the accumulation of fluoroquinolones by *Staphylococcus aureus*. *J. Antimicrob. Chemother.*, **2001**, *47*(3), 261-70.<br>Takenouchi, T.; Tabata, F.; Iwata, Y.; Hanzawa, H.; Sugawara, M.; Ohya, S. Hydrophilicity of quinolones is not an exclusive factor for decreased activity in efflux-mediated resistant mutants of *Staphylococcus aureus*. *Antimicrob. Agents Chemother.*, **1996**, *40*(8), 1835-42.<br>Mitsuyama, J.; Itoh, Y.; Takahata, M.; Okamoto, S.; Yasuda, T. *In vitro* antibacterial activities of tosufloxacin against and uptake of tosufloxacin by outer membrane mutants of *Escherichia coli*, *Proteus mirabilis*, and *Salmonella typhimurium*. *Antimicrob. Agents Chemother.*, **1992**, *36*(9), 2030-6.<br>Tanaka, M.; Yamazaki, E.; Chiba, M.; Yoshihara, K.; Akasaka, T.; Takemura, M.; Sato, K. *In vitro* antibacterial activities of DQ-113, a potent quinolone, against clinical isolates. *Antimicrob. Agents Chemother.*, **2002**, *46*(3), 904-8.<br>Gargallo-Viola, D.; Ferrer, S.; Tudela, E.; Robert, M.; Coll, R.; Roser, R, Guinea J. Antibacterial activities and pharmacokinetics of E-4767 and E-5065, two new 8-chlorofluoroquinolones with a 7-azetidin ring substituent. *Antimicrob. Agents Chemother.*, **2001**, *45*(11), 3113-21.

		Neu, H.C.; Chin, N.X. *In vitro* activity of the new fluoroquinolone CP-99,219. *Antimicrob. Agents Chemother.*, **1994**, *38*(11), 2615-22.<br>Neu, H.C.; Fang, W.; Gu, J.W.; Chin, N.X. *In vitro* activity of OPC-17116. *Antimicrob. Agents Chemother.*, **1992**, *36*(6), 1310-5.
Trovafloxacin	7-((1R,5S,6s)-6-amino-3-azabicyclo[3.1.0]hexan-3-yl)-1-(2,4-difluorophenyl)-6-fluoro-4-oxo-1,4-dihydro-1,8-naphthyridine-3-carboxylic acid	Lubasch, A.; Keller, I.; Borner, K.; Koeppe, P.; Lode, H. Comparative pharmacokinetics of ciprofloxacin, gatifloxacin, grepafloxacin, levofloxacin, trovafloxacin, and moxifloxacin after single oral administration in healthy volunteers. *Antimicrob. Agents Chemother.*, **2000**, *44*(10), 2600-3.<br>Andersson, M.I.; MacGowan, A.P. Development of the quinolones. *J Antimicrob Chemother.*, **2003**, *51*(S1), 1-11.<br>Dalvie, D.K.; Khosla, N.; Vincent, J. Excretion and metabolism of trovafloxacin in humans. *Drug Metab. Dispos.*, **1997**, *25*(4), 423-7.<br>Melnik, G.; Schwesinger, W.H.; Teng, R.; Dogolo, L.C.; Vincent, J. Hepatobiliary elimination of trovafloxacin and metabolites following single oral doses in healthy volunteers. *Eur. J. Clin. Microbiol. Infect. Dis.*, **1998**, *17*(6), 424-6.<br>Cormican, M.G.; Jones, R.N. Antimicrobial activity and spectrum of LB20304, a novel fluoronaphthyridone. *Antimicrob. Agents Chemother.*, **1997**, *41*(1), 204-11.<br>Goldstein, E.J.; Citron, D.M.; Hudspeth, M.; Hunt Gerardo, S.; Merriam, C.V. Trovafloxacin compared with levofloxacin, ofloxacin, ciprofloxacin, azithromycin and clarithromycin against unusual aerobic and anaerobic human and animal bite-wound pathogens. *J. Antimicrob. Chemother.*, **1998**, *41*(3), 391-6. Brueggemann, A.B.; Kugler, K.C.; Doern, G.V.

		In vitro activity of BAY 12-8039, a novel 8-methoxyquinolone,

		compared to activities of six fluoroquinolones against *Streptococcus pneumoniae*, *Haemophilus influenzae*, and *Moraxella catarrhalis*. *Antimicrob. Agents Chemother.*, **1997**, *41*(7), 1594-7.<br>Yun, H.J.; Min, Y.H.; Lim, J.A.; Kang, J.W.; Kim, S.Y.; Kim, M.J.; Jeong, J.H.; Choi, Y.J.; Kwon, H.J.; Jung, Y.H.; Shim, M.J.; Choi, EC. *In vitro* and *in vivo* antibacterial activities of DW286, a new fluoronaphthyridone antibiotic. *Antimicrob. Agents Chemother.*, **2002**, *46*(9), 3071-4.<br>Hoellman, D.B.; Kelly, L.M.; Jacobs, M.R.; Appelbaum, P.C. Comparative antianaerobic activity of BMS 284756. *Antimicrob. Agents Chemother.*, **2001**, *45*(2), 589-92.<br>Montanari, M.P.; Mingoia, M.; Varaldo, P.E. *In vitro* antibacterial activities of AF 3013, the active metabolite of prulifloxacin, against nosocomial and community Italian isolates. *Antimicrob. Agents Chemother.*, **2001**, *45*(12), 3616-22.<br>Neu, H.C.; Chin, N.X. *In vitro* activity of the new fluoroquinolone CP-99,219. *Antimicrob. Agents Chemother.*, **1994**, *38*(11), 2615-22.<br>Snydman, D.R.; Jacobus, N.V.; McDermott, L.A.; Ruthazer, R.; Goldstein, E.; Finegold, S.; Harrell, L.; Hecht, D.W.; Jenkins, S.; Pierson, C.; Venezia, R.; Rihs, J; Gorbach, S.L. *In vitro* activities of newer quinolones against bacteroides group organisms. *Antimicrob. Agents Chemother.*, **2002**, *46*(10), 3276-9.<br>Milatovic, D.; Schmitz, F.J.; Brisse, S.; Verhoef, J.; Fluit, A.C. *In vitro* activities of sitafloxacin (DU-6859a) and six other fluoroquinolones against 8,796 clinical bacterial isolates. *Antimicrob. Agents Chemother.*, **2000**, *44*(4), 1102-7.<br>Almer, L.S.; Hoffrage, J.B.; Keller,

		E.L.; Flamm, R.K.; Shortridge, V.D. *In vitro* and bactericidal activities of ABT-492, a novel fluoroquinolone, against Gram-positive and Gram-negative

	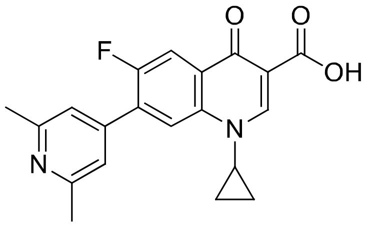	organisms. *Antimicrob. Agents Chemother.*, **2004**, *48*(7), 2771-7.<br>Nilius, A.M.; Shen, L.L.; Hensey-Rudloff, D.; Almer, L.S.; Beyer, J.M.; Balli, D.J.; Cai, Y.; Flamm, R.K. *In vitro* antibacterial potency and spectrum of ABT-492, a new fluoroquinolone. *Antimicrob. Agents Chemother.*, **2003**, *47*(10), 3260-9.<br>Jones, R.N.; Croco, M.A.; Pfaller, M.A.; Beach, M.L.; Kugler, K.C. Antimicrobial activity evaluations of gatifloxacin, a new fluoroquinolone: contemporary pathogen results from a global antimicrobial resistance surveillance program (SENTRY, 1997). *Clin. Microbiol. Infect.*, **1999**, *5*(9), 540-6.<br>Felmingham, D.; Robbins, M.J.; Ingley, K.; Mathias, I.; Bhogal, H.; Leakey, A.; Ridgway, G.L.; Grüneberg, R.N. *In-vitro* activity of trovafloxacin, a new fluoroquinolone, against recent clinical isolates. *J. Antimicrob. Chemother.*, **1997**, *39*(SB), 43-9.<br>Cunha, B.A.; Qadri, S.M.; Ueno, Y.; Walters, E.A.; Domenico, P. Antibacterial activity of trovafloxacin against nosocomial Gram-positive and Gram-negative isolates. *J. Antimicrob. Chemother.*, **1997**, *39*(SB), 29-34.<br>Endtz, H.P.; Mouton, J.W.; den Hollander, J.G.; van den Braak, N.; Verbrugh, H.A. Comparative *in vitro* activities of trovafloxacin (CP-99,219) against 445 gram-positive isolates from patients with endocarditis and those with other bloodstream infections. *Antimicrob. Agents Chemother.*, **1997**, *41*(5), 1146-9.<br>Barry, A.L.; Fuchs, P.C.; Brown, S.D. *In vitro* activities of three nonfluorinated quinolones against representative

		bacterial isolates. *Antimicrob. Agents Chemother.*, **2001**, *45*(6), 1923-7.<br>Gooding, B.B.; Jones, R.N. *In vitro* anti-microbial activity of CP-99,219, a novel azabicyclo-naphthyridone.<br>*Antimicrob. Agents Chemother.*, **1993**, *37*(2), 349-53.
WIN57273	1-cyclopropyl-7-(2,6-dimethylpyridin-4-yl)-6-fluoro-4-oxo-1,4-dihydroquinoline-3-carboxylic acid	Michéa-Hamzehpour, M.; Furet, Y.X.; Pechère, J.C. Role of protein D2 and lipopolysaccharide in diffusion of quinolones through the outer membrane of *Pseudomonas aeruginosa*. *Antimicrob. Agents Chemother.*, **1991**, *35*(10), 2091-7.<br>Eliopoulos, G.M.; Klimm, K.; Rice, L.B.; Ferraro, M.J.; Moellering, R.C. Jr. Comparative *in vitro* activity of WIN 57273, a new fluoroquinolone antimicrobial agent. *Antimicrob. Agents Chemother.*, **1990**, *34*(6), 1154-1159.<br>Jones, R.N.; Barry, A.L. *In vitro* evaluation of WIN 57273, a new broad-spectrum fluoroquinolone.<br>*Antimicrob. Agents Chemother.*, **1990**, *34*(2), 306-13.<br>Sedlock, D.M.; Dobson, R.A.; Deuel, D.M.; Lesher, G.Y.; Rake, J.B. *In vitro* and *in vivo* activities of a new quinolone, WIN 57273, possessing potent activity against gram-positive bacteria. *Antimicrob. Agents Chemother.K,* **1990**, *34*(4), 568-75.
